# *Fensomea setacea*, gen. & sp. nov*.* (Cladopyxidaceae, Dinophyceae), is neither gonyaulacoid nor peridinioid as inferred from morphological and molecular data

**DOI:** 10.1038/s41598-021-92107-0

**Published:** 2021-06-17

**Authors:** Marc Gottschling, Maria Consuelo Carbonell-Moore, Kenneth Neil Mertens, Monika Kirsch, Malte Elbrächter, Urban Tillmann

**Affiliations:** 1 Department Biologie: Systematik, Biodiversität & Evolution der Pflanzen, GeoBio-Center, Ludwig‐Maximilians-Universität München, Menzinger Str. 67, 80 638 München, Germany; 2 Department of Botany and Plant Pathology, College of Agricultural Sciences, Oregon State University, 2082 Cordley Hall, Corvallis, OR 97331-2902 USA; 3 Ifremer, LITTORAL, Station de Biologie Marine, Place de la Croix, BP40537, 29900 Concarneau CEDEX, France; 4 Fachbereich Geowissenschaften-Fachrichtung Historische Geologie/Paläontologie, Universität Bremen, Klagenfurter Straße, 28359 Bremen, Germany; 5 Alfred-Wegener-Institute, Helmholtz Centre for Polar and Marine Research, Sylt, Hafenstr. 43, 25 992 List/Sylt, Germany; 6 Alfred-Wegener-Institute, Helmholtz Centre for Polar and Marine Research, Am Handelshafen 12, 27 570 Bremerhaven, Germany

**Keywords:** Evolution, Ocean sciences

## Abstract

Dinophyte evolution is essentially inferred from the pattern of thecal plates, and two different labelling systems are used for the important subgroups Gonyaulacales and Peridiniales. The partiform hypotheca of cladopyxidoid dinophytes fits into the morphological concepts of neither group, although they are assigned to the Gonyaulacales. Here, we describe the thecate dinophyte *Fensomea setacea*, gen. & sp. nov*.*, which has a cladopyxidoid tabulation. The cells displayed a Kofoidean plate formula APC, 3′, 4a, 7″, 7C, 6S, 6′′′, 2′′′′, and slender processes were randomly distributed over the echinate or baculate surface. In addition, we obtained rRNA sequences of *F. setacea*, gen. & sp. nov*.*, but dinophytes that exhibit a partiform hypotheca did not show a close relationship to Gonyaulacales. Character evolution of thecate dinophytes may have progressed from the ancestral state of six postcingular plates, and two more or less symmetrically arranged antapical plates, towards patterns of only five postcingular plates (Peridiniales) or more asymmetrical configurations (Gonyaulacales). Based on our phylogenetic reconsiderations the contact between the posterior sulcal plate and the first postcingular plate, as well as the contact between an antapical plate and the distalmost postcingular plate, do not represent a rare, specialized gonyaulacoid plate configuration (i.e., the partiform hypotheca of cladopyxidoid dinophytes). Instead, these contacts correspond to the common and regular configuration of peridinioid (and other) dinophytes.

## Introduction

Over time, evolution has produced impressive biodiversity in the world’s oceans, including multicellular organisms, such as animals, and unicellular organisms that make significant ecological contributions^1,2^. Diatoms, foraminifers, and radiolarians are iconic unicellular groups, in which our knowledge of their biodiversity is far from complete. Another key group of planktonic organisms are the Dinophyceae, which have great ecological and economic importance because of their different nutrition types and the toxicity of a considerable number of species^3–5^. During their life-history, many dinophytes undergo different developmental stages that usually include a motile, flagellated stage and a coccoid stage broadly interpreted as resting and/or dormancy cells (or ‘cysts’)^6–8^. Many dinophytes also have a cell wall made up of cellulose plates (collectively, the theca) that has a pattern that may be group or species specific.

Many Dinophyceae are more or less spherical and have a smooth surface, but there are exceptions, including groups that form wings (e.g., Dinophysales) or thorny processes (e.g., Ceratiaceae)^9^. One particular species, *Cladopyxis brachiolata* F.Stein, is a dinophyte with exceptional morphology because of the presence of long and robust thecal processes^10–12^. The original species description by Friedrich von Stein^10^ was accompanied by drawings (here reproduced as Fig. S1). These drawings illustrate cells with heterogeneous morphologies and thecal processes that vary in number and shape, which can be either long or short and distally undivided, bifurcate, or multi-branching. This morphological distinctiveness is at the base of the early recognition of *Cladopyxis* F.Stein as a taxon at the family rank^10^. The Cladopyxidaceae (Dinophyceae) comprise small microalgae distributed in marine, (sub)tropical habitats^8,13^. They are morphologically well circumscribed but to this day, knowledge of their life-history and molecular characteristics is still very limited. Cladopyxidoid dinophytes are an important presence in the fossil record (including groups such as †Lotharingiaceae, †Mancodiniaceae, †Pareodiniaceae, and †Scriniocassiaceae), particularly during the Jurassic–Cretaceous, but are uncommon in the Neogene^8,14–16^. No unequivocal fossils are known from the Quaternary.

**Figure 1:**
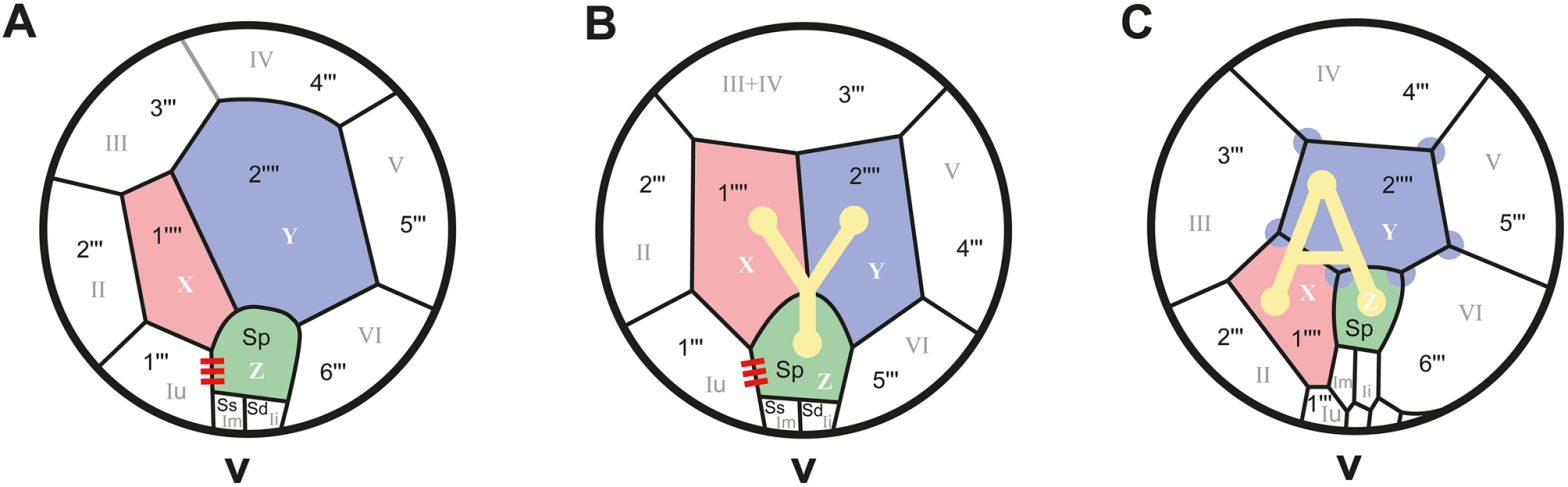
Schematics of hypothecae in armoured dinophytes. (**A**) Cladopyxidoid, (**B**) peridinialean, (**C**) gonyaulacalean: sexiform (modified^8^). Putatively homologous plates are color-coded, using the AY-model system^17^. For plate labelling, we followed Kofoidean notation^23^ (black lettering), while the Taylor-Evitt notation is in grey. Thus, our first antapical plate corresponds to a posterior intercalary plate, and our second antapical plate corresponds to the only antapical plate^3,8,17,18^. Note the (putatively ancestral) connection between the posterior sulcal plate and the first postcingular plate (red triple bar in **A** and **B**). The reduction from six to five postcingular plates in Peridiniales may result from a fusion of the third and fourth postcingular plates as they are present today in cladopyxidoid dinophytes including *Fensomea setacea*, gen. & sp. nov*.*

During the 1970s, detailed studies of the thecal plate patterns of dinophytes were carried out, which resulted in the erection of the Gonyaulacales and its systematic delimitation from the Peridiniales^17^. The most conserved diagnostic characteristic is the presence of six postcingular plates in Gonyaulacales versus five such plates in Peridiniales. Relative to the flagellar insertion, the hypotheca shows an ‘A’-shaped arrangement of antapical plates in gonyaulacoid ancestors and a ‘Y’-shaped arrangement in hypothetical peridinioid ancestors^3,8,17,18^ (Fig. 1B,C). Both groups exhibit two plates in the antapical region of the cell; one of these plates is considered the posterior intercalary plate for Gonyaulacales by some^19,20^. Moreover, the posterior sulcal plate abuts the first postcingular plate in Peridiniales, whilst this contact is usually absent in Gonyaulacales.

Four basic types of hypothecal plate arrangements are readily distinguished in Gonyaulacales: corniform; sexiform (Fig. 1C); quinqueform; and partiform (Fig. 1A)^8,18^. The corniform and quinqueform types represent internally derived character states in the Gonyaulacales^21^ and were not considered in this study. The sexiform hypotheca represents the ancestral character state for Gonyaulacales present in a paraphyletic grade, but it is the partiform hypotheca that was considered rare and exclusive to the Cladopyxidaceae in extant dinophytes^8,18^.

Using Kofoidean^22^ and Taylor-Evitt^3,8,17,18^ notations, two alternative plate labelling systems were developed for dinophytes with each claiming to account for plate homologies. Using the Taylor-Evitt notation (relevant particularly for gonyaulacoid dinophytes), the partiform hypotheca can be described by the Y plate adjacent to the most distal postcingular homologue (VI plate) and by the Z plate consistently located within the sulcus. The Z plate is commonly omegaform (i.e., broader towards its antapical end). It reaches further anterior than the X plate (or assumed posterior intercalary plate), consequently contacting the first postcingular homologue (Iu plate). The large size of the Z plate, and its anterior extension, is reciprocated by the small size of the left and right sulcal plates (i.e., Ii, Im).

A considerable number of previous studies did not follow the Taylor-Evitt notation and applied Kofoidean labelling to peridiniod and gonyaulacoid dinophytes. The plates of the hypotheca adjacent to the sulcus but that do not contact the cingulum are defined as antapical plates^23^. Based on this definition, and in accordance with Kofoidean labelling, a partiform arrangement of the hypotheca is circumscribed by (a) the presence of six postcingular plates, (b) the second antapical plate (2′′′′) contacting six adjacent plates and, in particular, the most distal postcingular plate (6′′′), and (c) the contact between the large posterior sulcal plate (Sp) and the first postcingular plate (1′′′). In this circumscription, a partiform hypotheca (Fig. 1C) is certainly present in *Cladopyxis*^12^. Other extant dinophytes exhibiting partiform hypotheca are the Amphidomataceae^24^, but their possible relationship to Cladopyxidaceae has not been clearly worked out at present.

The distinctiveness between Gonyaulacales and Peridiniales lineages has been confirmed by molecular phylogenetics^25–29^. However, taxa with available DNA sequence information and a partiform hypotheca (i.e., Amphidomataceae) do not show clear phylogenetic proximity to either of these two major dinophyte lineages. Thus, extant Cladopyxidaceae may provide a missing link of thecate dinophytes that would enable a better understanding of the first evolutionary transformations from ancestral configurations towards the more abundant and derived patterns in Gonyaulacales and Peridiniales^15,26,30–32^. To investigate this potential link, as well as the taxonomy of the constituent elements of the Cladopyxidaceae, the morphology of the thecal plate pattern and phylogenetic placement must be determined. In this study, we elucidate new steps towards achieving this integrative goal and describe a new cladopyxidoid dinophyte. We present light and scanning electron microscopy (LM and SEM, respectively) results of cultivated material and discuss our findings with regards to previous SEM studies of Cladopyxidaceae^33,34^. We combine these morphological data with the first DNA sequence data of a cladopyxidoid dinophyte.

## Results

### Strain GeoB*184 morphology

**Figure 2:**
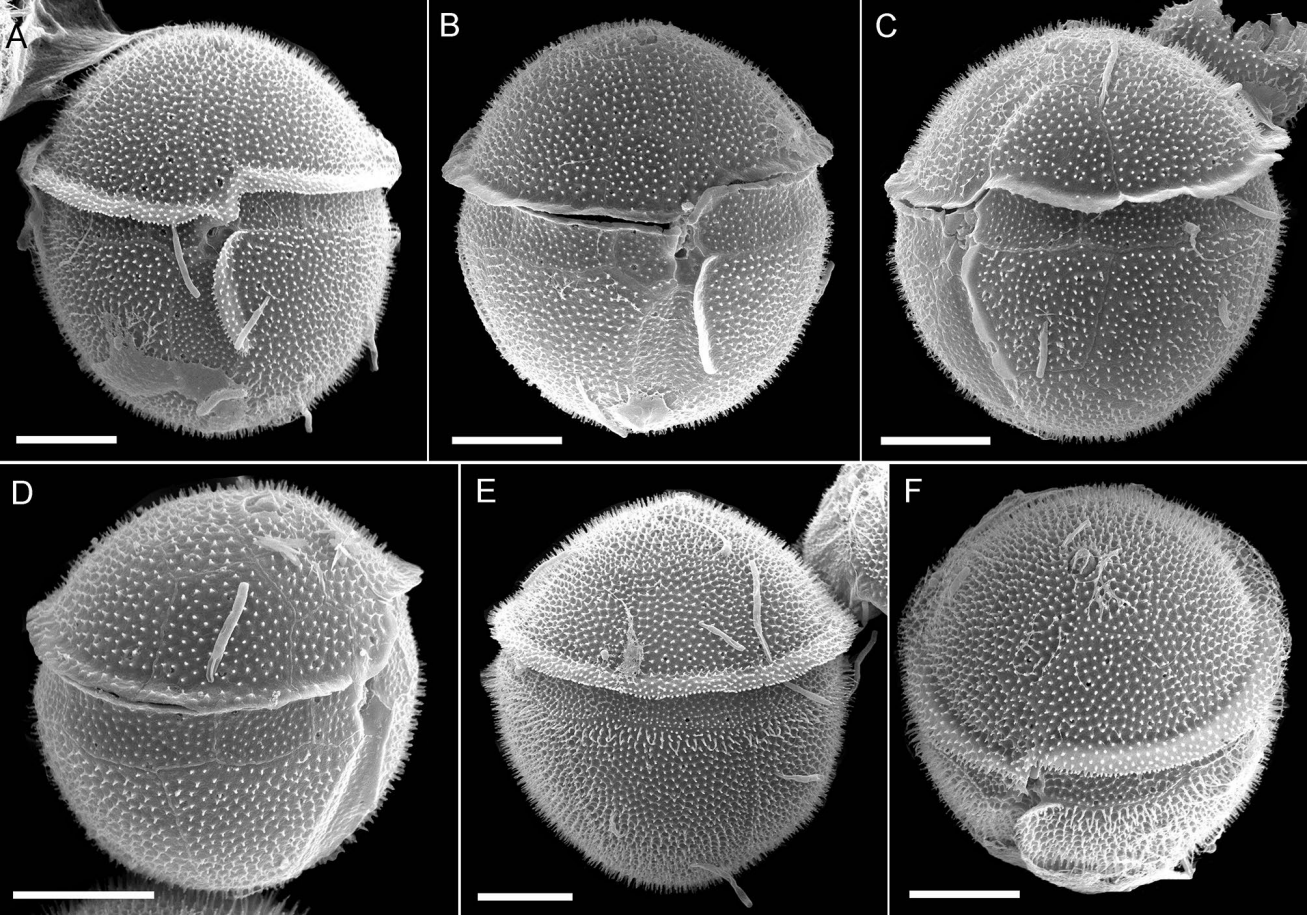
Scanning electron micrographs of *Fensomea setacea*, gen. & sp. nov*.* (GeoB*184) in (**A**, **B**) ventral view, (**C**) left lateral view, (**D**) right lateral view, (**E**) dorsal view, and (**F**) ventral apical view. Scale bars = 5 µm.

Phototrophic cells of GeoB*184 were brownish in colour and subspherical through oval in outline, without dorsoventral compression (Fig. 2). The hyposome was slightly longer than the episome and therefore, the broad cingulum (ca 2–4 µm in width) was pre-median in position. The descending cingulum was displaced by half of its width (Fig. 2A,B), scarcely incised, and had a distinct cingular list present only along its apical suture (Fig. 2). Cell size (as estimated from SEM images) was 19.8 µm in length (± 1.9 SD, range 13.8–23.3 µm, n = 32) and 18.0 µm in width (± 1.4 SD, range 13.5–21.2 µm, n = 32). The surface of all plates, except for some small sulcal plates, was echinate through baculate (i.e., densely covered by acuminate, elongate spines; density: 6.1 ± 0.1 spines µm^−2^, range 5.0–7.5, n = 21). Spine length was variable and usually ranged between 0.3 and 0.8 µm (mean 0.4 ± 0.1, n = 20). Plate sutures were difficult to ascertain but for some cells, spines were exceptionally reduced to small pimples (i.e., rounded elements), which allowed for a clearer view of the cell’s surface (Fig. 4A,G–I).

**Figure 3:**
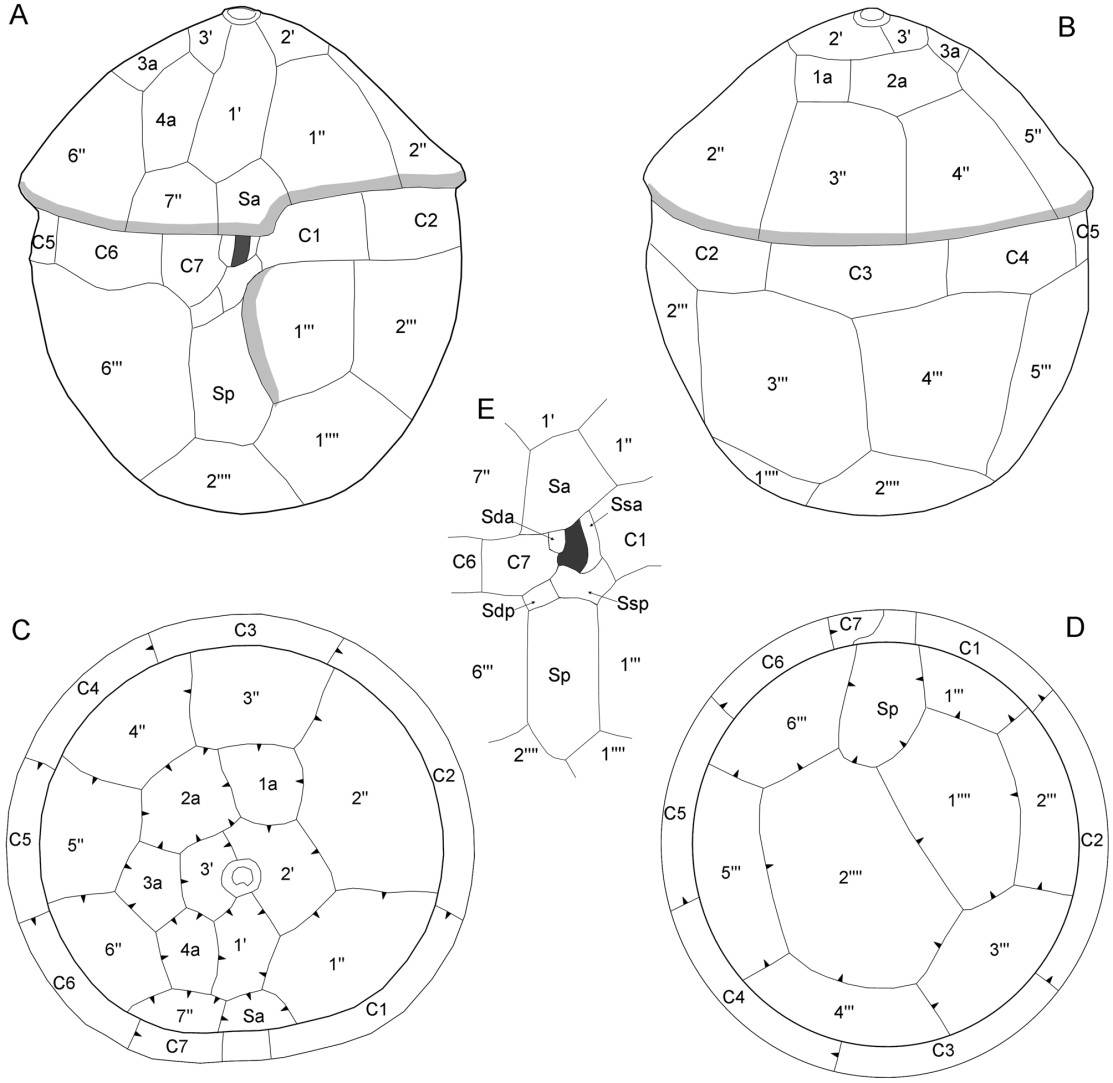
Schematic thecal plate pattern of *Fensomea setacea*, gen. & sp. nov. (**A**) Ventral, (**B**) dorsal, (**C**) apical, and (**D**) antapical views, and (**E**) details of the sulcal area. Plate labels according to the Kofoidean system. The grey-shading in (**A**) and (**B**) denote cingular and sulcal lists. The dark shading in the central part in (**A**) and (**E**) denote the presumed area from which both flagella emerge. The arrowheads in (**C**) and (**D**) indicate the direction of plate overlap. Abbreviation of sulcal plates: *Sa* anterior sulcal, *Sda* right anterior sulcal, *Sdp* right posterior sulcal, *Sp* posterior sulcal, *Ssa* left anterior sulcal, *Ssp* left posterior sulcal.

**Figure 4:**
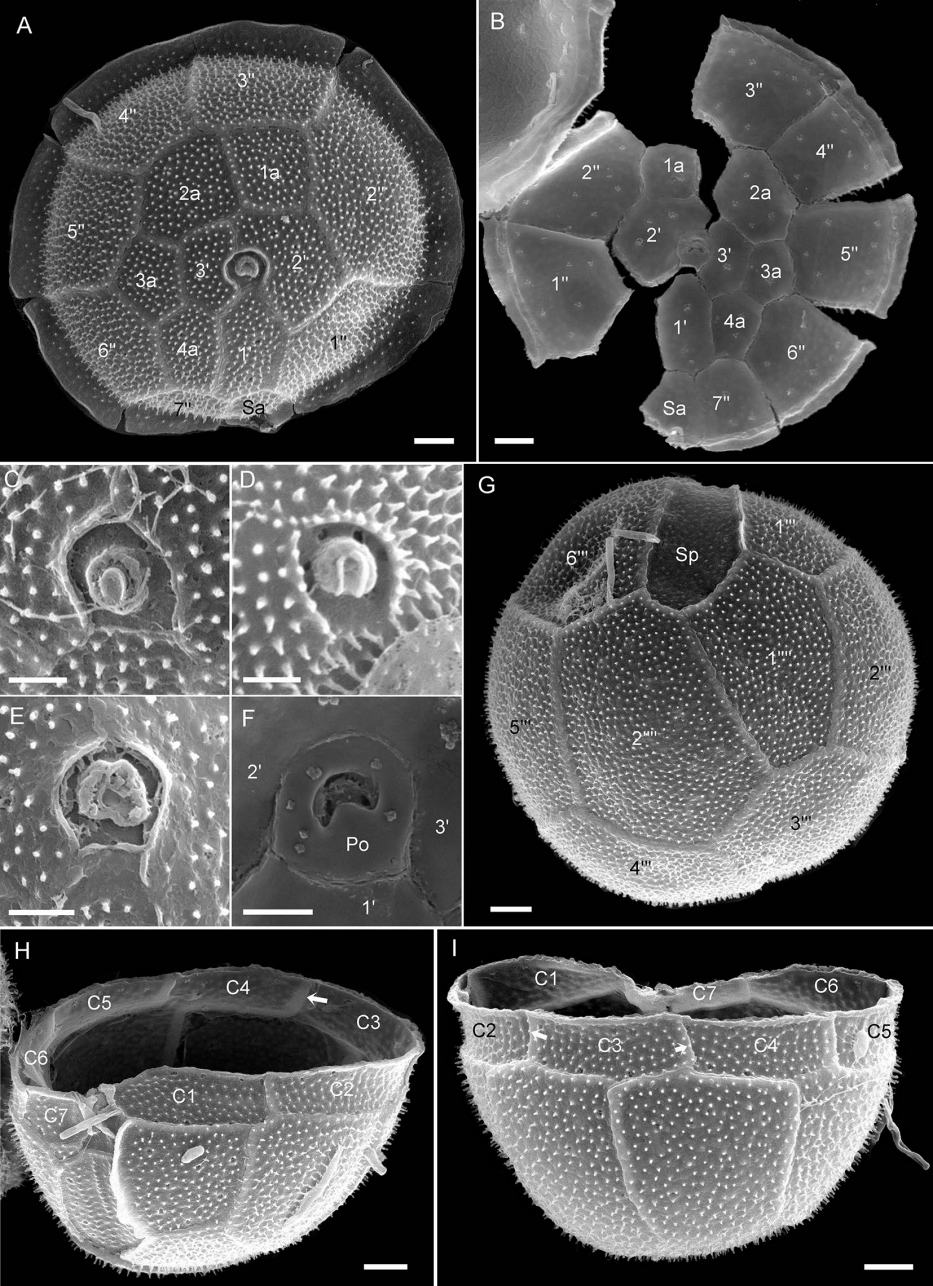
Scanning electron micrographs of *Fensomea setacea*, gen. & sp. nov*.* (GeoB*184). (**A**, **B**) Epitheca from (**A**) external and (**B**) internal views, (**C**–**F**) detailed view of the apical pore complex externally (**C**–**E**) or internally (**F**), (**G**) hypotheca in antapical view, (**H**–**I**) hypotheca in (**H**) ventral lateral view or (**I**) in dorsal view to indicate cingular plates. Scale bars = 2 µm (**A**, **B**, **G**–**I**) or 1 µm (**C**–**F**).

The plate formula was APC, 3′, 4a, 7″, 7C, 6S, 6′′′, 2′′′′ and is schematically drawn in Fig. 3. The epitheca (Fig. 4A,B) consisted of three apical plates, four anterior intercalary plates, seven precingular plates, and an apical pore complex (APC). The first apical plate was broad, heptagonal, and rectangular in the centre. The second apical plate was heptagonal and larger than the hexagonal plate 3′. The four anterior intercalary plates formed a series (dorsal to ventral) on the cell’s right side. Plate 1a was square, whereas the larger and irregularly shaped plate 2a was heptagonal. Both plates 3a and 4a were pentagonal, with plate 4a being more elongated and located ventrally next to plate 1′. Each of the precingular plates was in contact with four other epithecal plates (including plate Sa), except for plate 4″, which was adjacent to three other epithecal plates. The first two precingular plates were slightly broader compared to the remaining plates of the series; plate 7″ was the smallest precingular plate (Fig. 4A,B). The APC (Fig. 4C–F) consisted of a rounded pore plate with a straight or slightly curved ventral suture with plate 1′. The pore plate was bordered on its dorsal and lateral sides by a minute elevated rim formed by the sutures of plates 2′ and 3′. In the centre of the pore plate, there was a small and slightly raised tubular or globular structure with a horseshoe-shaped cover plate (cp), which was generally obscured by mucus and difficult to observe. Internal views of the pore plate (Fig. 4F) revealed a crescent-shaped apical pore opening.

When disregarding the sulcal plates, the partiform hypotheca consisted of six postcingular and two antapical plates (Fig. 4G). Each postcingular plate was in contact with three other hypothecal plates, with the exception of plate 3′′′ that was adjacent to both antapical plates and thus to four other hypothecal plates. All postcingular plates were similar in width, except for plate 1′′′, which was narrower. The right side of this plate bore a distinct, curved flange that partly covered the sulcal area. The antapical plates were dissimilar in size; plate 1′′′′ was smaller, pentagonal, and positioned slightly more ventral compared to the hexagonal plate 2′′′′ (Fig. 4G). The cingulum (Fig. 4H,I) was composed of seven cingular plates of almost equal size; plate C7 was distinctly smaller. The posterior cingular suture was zigzag-shaped (most obvious on pentagonal dorsal plates C3 and C4, less distinctive on plate C1).

**Figure 5:**
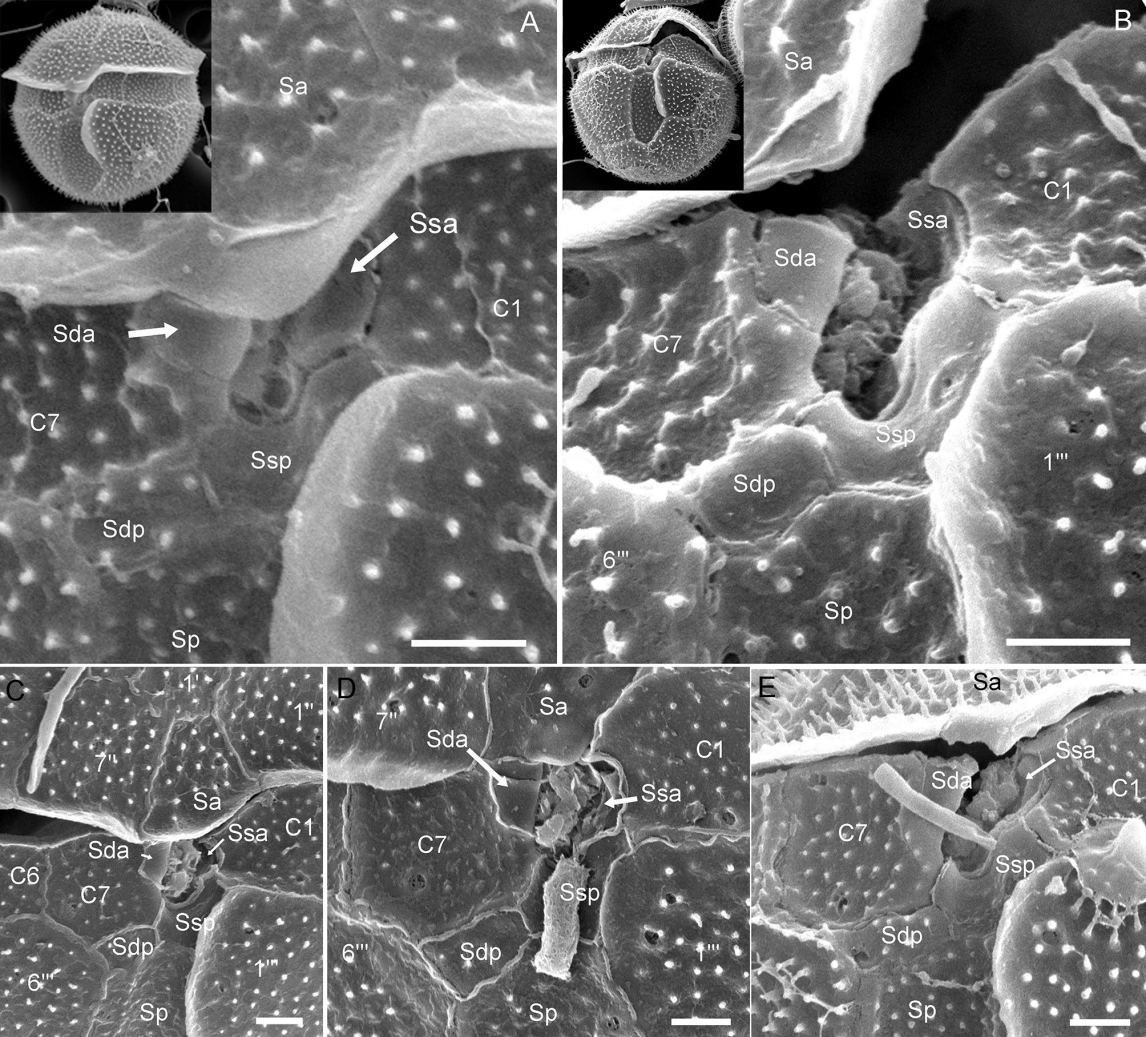
Scanning electron micrographs of *Fensomea setacea*, gen. & sp. nov*.* (GeoB*184). (**A**–**E**) Detailed view of the sulcal area. Abbreviation of sulcal plates: *Sa* anterior sulcal, *Sda* right anterior sulcal, *Sdp* right posterior sulcal, *Sp* posterior sulcal, *Ssa* left anterior sulcal, *Ssp* left posterior sulcal. Scale bars = 1 µm.

Six sulcal plates were identified (Figs. 3E, 5). The anterior sulcal plate (Sa) was part of the epitheca (Fig. 4A,B). The posterior sulcal plate (Sp) was large, at least twice as long as wide, and extended posteriorly almost to the antapex (Figs. 2, 4G). Anterior to plate Sp, there were two small sulcal plates (right and left posterior sulcal plates: Sdp and Ssp, respectively). Plate Ssp formed the posterior, emarginate area, where presumably both flagella emerged (Fig. 3E), and was adjacent to both the first (C1) and last (C7) cingular plates. Two small anterior plates (Sda and Ssa) were arranged lateral to the flagellar opening. These two plates as well as plate Ssp (but not plate Sdp) did not bear ornamentation (Fig. 5). In strain GeoB*184, deviations from this plate pattern were occasionally observed, including the fusion or subdivision of epithecal and/or hypothecal plates (Fig. S2).

**Figure 6:**
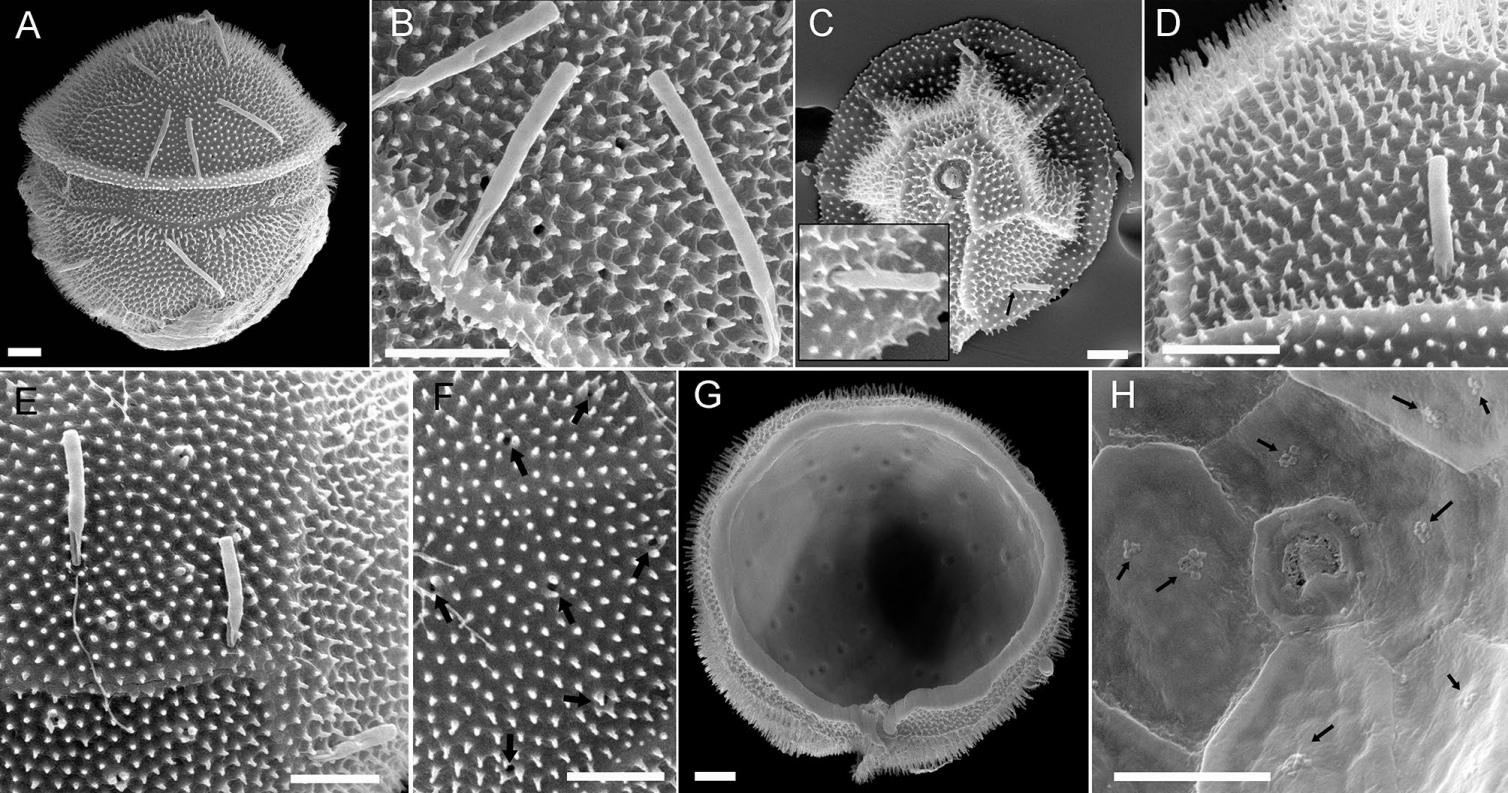
Scanning electron micrographs of *Fensomea setacea*, gen. & sp. nov*.* (GeoB*184), showing detailed views of (**A**–**E**) slender processes and (**E**–**H**) pore-like structures (black arrows) in the plates. Note the bipolar differentiation of the processes, which have striate proximal ends and glabrous distal ends. Scale bars = 2 µm.

**Figure 7:**
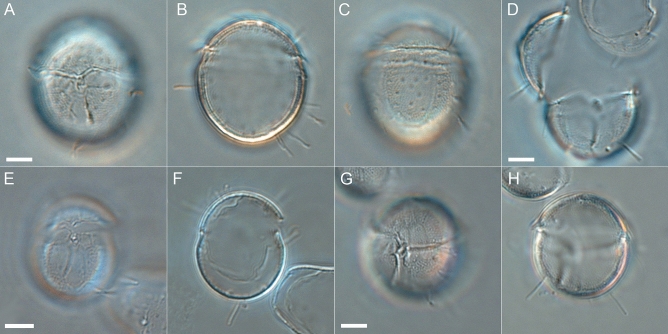
Light microscopy images of *Fensomea setacea*, gen. & sp. nov. (GeoB*184), that illustrate the echinate through baculate ornamentation and the slender processes. (**A**–**C**) Different focal planes of a cell in ventral (**A**), median (**B**) or dorsal view (**C**). (**D**) Cell in ventral view; note that the epitheca has broken up. (**E**, **F**) Two different focal planes of a cell in dorsal view. (**G**, **H**) Two focal planes of a cell in ventral-lateral view. Scale bars = 5 µm.

In SEM preparations of strain GeoB*184, slender processes (‘setae’) were scattered over the cell’s surface. The setae proximal surfaces were striate, and distal surfaces were glabrous (Figs. 2, 6A–E). Their diameter was consistent (0.36 ± 0.02 µm, n = 32), but length varied from 1.9 to 6.3 µm (mean 3.8 ± 1.2, n = 27). In most cases, these structures seemed to be randomly arranged and not regularly attached to the cells but occasionally, they appeared to be attached to small pores (Fig. 6C,D). Moreover, depressions with round outer edges were visible on both epithecal (including the pore plate) and hypothecal plates, which were characteristically surrounded by three or four tiny granules (Fig. 6B,E,F). It was unclear whether these structures penetrated the plates like true pores. Internally, the position of these structures in the hypo- and epitheca (Figs. 4F, 6G,H) was visible not as openings but as small bumps or margined by minute granular depositions. The slender processes and the echinate cell surface were also seen under LM (Fig. 7).

The presence of growth bands, interior thecal views (e.g., Figs. 4B, 5G,H), and thecate cells with slightly disarranged plates allowed for identification regarding the overlap pattern of plate margins (Fig. 3C,D). Keystone plates (i.e., those plates overlapping all of their neighbours) from the cingular, precingular, and postcingular series were plates C3, 3″, and 4′′′, respectively. Plate 2a was overlapped by all adjacent plates, which was a notable trait of the epitheca. However, the overlap pattern of the sulcal plates could not be elucidated.

### Molecular phylogenetics

**Figure 8:**
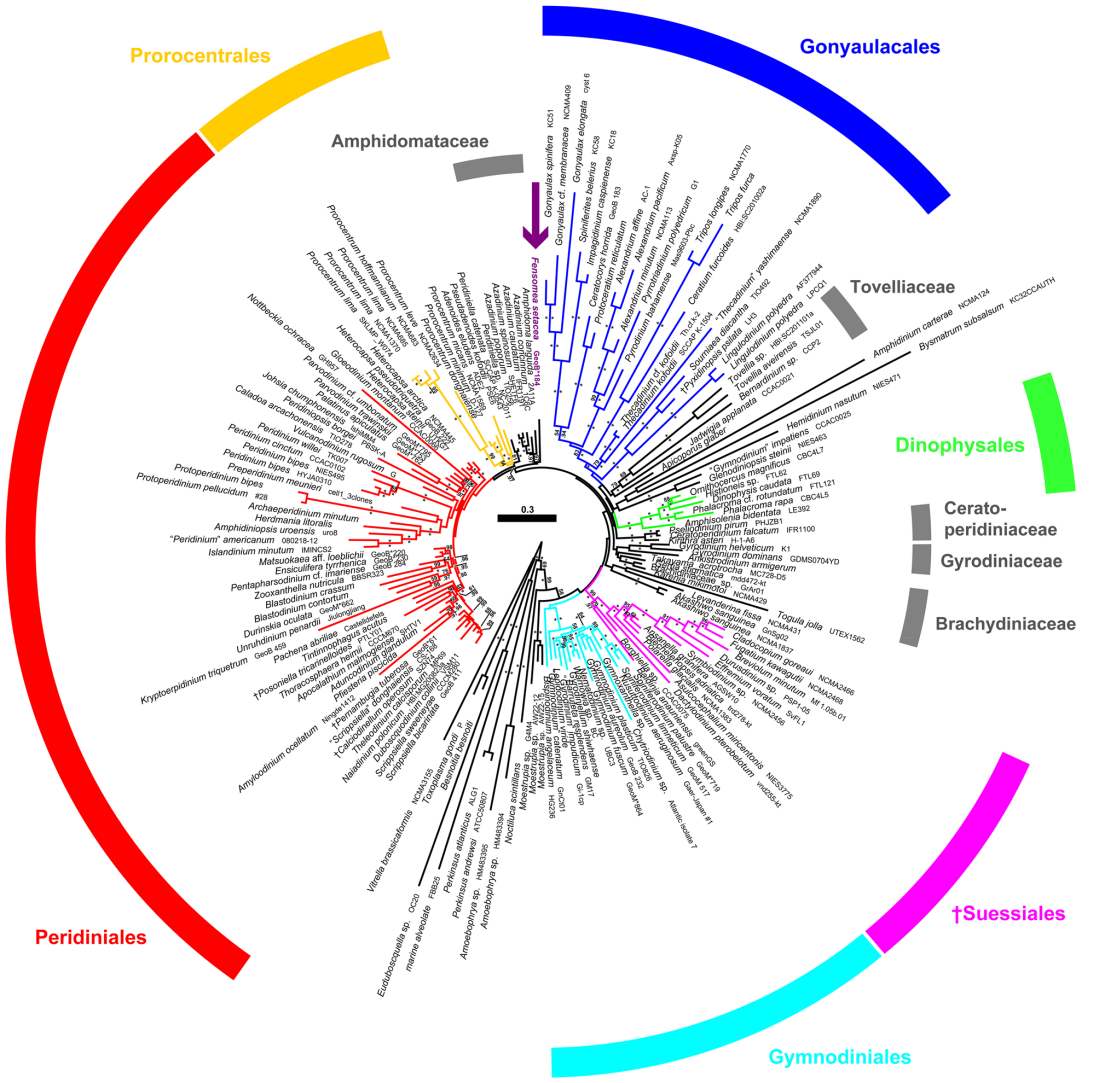
A molecular reference tree recognizing major groups of dinophytes. Maximum Likelihood (ML) tree of 161 systematically representative dinophyte sequences (with strain number information) inferred from a rRNA nucleotide alignment (3632 parsimony-informative positions). The numbers on the branches are ML bootstrap (above the branch line) and Bayesian probabilities (below the branch line) for the clusters (asterisks indicate maximal support values; values under 50 for the ML bootstrap and 0.90 for Bayesian probability are not shown). Note the phylogenetic position of *Fensomea setacea*, gen. & sp. nov*.*, which indicates it cannot be clearly assigned to Gonyaulacales or Peridiniales.

The rRNA (i.e., SSU + ITS + LSU) reference alignment was 1871 + 2076 + 3753 bp long and comprised 825 + 1044 + 1763 parsimony informative sites (42.0%, mean of 22.6 per terminal taxon) and 5498 distinct RAxML alignment patterns. Tree topologies were largely congruent, irrespective of whether the Bayesian or ML algorithm was applied. Figure 8 shows the best-scoring ML tree (− ln = 186,512.02), with the internal topology not fully resolved. However, Dinophyceae were monophyletic (99LBS, 1.00BPP) and many nodes were statistically well, if not maximally, supported. A number of lineages at high taxonomic level, such as Dinophysales (100LBS, 1.00BPP), Gonyaulacales, Gymnodiniales (excl. *Bispinodinium* N.Yamada & T.Horig.: 100LBS, 1.00BPP), Peridiniales, Prorocentrales (0.97BPP), and †Suessiales (99LBS, 1.00BPP), as well as Amphidomataceae (97LBS, 0.97BPP), Brachydiniaceae, Ceratoperidiniaceae (100LBS, 1.00BPP), Gyrodiniaceae (100LBS, 1.00BPP), and Tovelliaceae (79LBS, 1.00BPP) were recognized. Only 17 of 161 dinophyte accessions (10.6%), scattered over the tree, were not assigned to any of those lineages, including *F. setacea*, gen. & sp. nov., which had an unresolved phylogenetic position (seemingly close to the Amphidomataceae, but without support).

## Discussion

### Diversity of thecal processes

Planktonic cells with elongated processes are rare among extant dinophytes, and three types can be readily distinguished: (a) delicate, unbranched, and filiform setae of *Micracanthodinium* Deflandre; (b) robust, striated, and intratabular (unbranched and branched) processes present in *Acanthodinium* Kof. and *Cladopyxis*; (c) slender processes (visible even under LM) that are randomly distributed over the cell surface and not associated with particular thecal plates. To the best of our knowledge, *F. setacea*, gen. & sp. nov*.*, is unique in exhibiting this final characteristic (c), but whether these setae are present in all stages of life-history, as well as their precise function, remains to be determined.

The setae described here were present on cells derived from cultivated material, but they have previously been documented in field samples^34^; thus, a culture artefact appears unlikely. In a few cases, ambient conditions, such as temperature, salinity, or turbulence, have been shown to modify surface features and process length in coccoid cells of extant dinophytes^35–37^. However, this has not been demonstrated in thecate cells. Additionally, there are no data that show long, robust, and divided processes can become short, fine, and unbranched as a result of culture conditions. Thus, we consider the slender processes of *F. setacea*, gen. & sp. nov*.*, to be a stable feature and the most striking diagnostic trait to delimit our new taxon from previously described species (see “Taxonomic activity”). This characteristic also delimits *F. setacea*, gen. & sp. nov*.*, from other extant cladopyxidoid taxa without processes, such as *Palaeophalacroma* J.Schiller (= *Epiperidinium* Gaarder) and *Sinodinium* D.S.Nie^12,38^ (which also have no partiform hypotheca).

The presence of thecal plates was not noted in any of the original descriptions of species currently assigned to *Micracanthodinium*. In a subsequent SEM study^34^ that claimed to illustrate the tabulation pattern of *Micracanthodinium* for the first time, no rigorous explanation for the identification of *Micracanthodinium setiferum* (Lohmann) Deflandre was provided, and two different organisms may have been studied (compare his Figs. 2 and 6). As a result, there is still no published study that reliably shows the filiform setae of true *Micracantho-dinium* together with a dinophyte plate pattern. Thus, it remains unclear whether the SEM plates of John D. Dodge^34^ include cells assignable to *F. setacea*, gen. & sp. nov.

There is an open question whether all such setae and processes that are variously slender, robust, or branched are homologous among dinophytes. In particular, it is not even known at present whether the setae of true *Micracantho-dinium* (distributed mainly along the cingulum margins) conform with ‘skeleton’-based structures (as in *Acanthodinium* and *Cladopyxis*, in which they appear associated with specific thecal plates) or ‘membrane’-based structures, such as pseudopodia. It is likely that at least the robust and smaller processes of *C. hemibrachiata* and Balech *F. setacea*, gen. & sp. nov*.*, respectively, are homologous because their plate patterns are very similar (see “Epithecal configurations”). Thus, *Acanthodinium*, *Cladopyxis*, and *F. setacea*, gen. & sp. nov., may appear as integral elements of the Cladopyxidaceae, but the taxonomic identity of *Micracanthodinium* from its type locality in Sicily^39^, and its relationship to cladopyxidoid^40,41^ or other (last not least unarmoured) dinophytes, remains elusive.

### Hypothecal configurations

The two major branches of dinophytes, Gonyaulacales and Peridiniales, present a mosaic combination of ancestral and derived character states. Despite the small number of extant species, cladopyxidoid protists are important for evolutionary interpretations because their seemingly rare plate pattern allows character polarity to be identified^15,26,31,32^. The precise systematic position of Cladopyxidaceae within the Dinophyceae, their internal taxonomic delimitations, and the phylogenetic relationships between their constituent elements have not sufficiently worked out. Accurate interpretations of the thecal plate pattern, and homologies between plates, are key to the development of a consistent evolutionary scenario. Charles A. Kofoid was the first person to interpret a cladopyxidoid tabulation for *Acanthodinium spinosum* Kof. and *C. brachiolata* (= *Acanthodinium caryophyllum* Kof.)^42^, but the plates were not yet labelled using his Kofoidean system because he developed that later^22^. However, the drawings show good congruence with later interpretations, particularly *C. brachiolata*^12^. Based on such studies *Cladopyxis* and putative relatives, including *F. setacea*, gen. & sp. nov*.*, have thecal series comprising three apical, three or four anterior intercalary^12^, seven precingular, six postcingular, and two antapical plates.

Despite his erection of the Gonyaulacales, Frank J.R. ‘Max’ Taylor regarded Cladopyxidaceae as phylogenetically closer to peridinioid dinophytes^17^. In contrast, William R. Evitt considered their plate pattern to be derived from sexiform gonyaulacoids rather than peridinioids^18^. However, *F. setacea*, gen. & sp. nov*.*, is not an integral part of the Gonyaulacales based on the DNA tree and instead represents an independent lineage within the dinophytes whose closest relatives cannot be ascertained reliably at present. This agrees with phylogenetic sketches (Fig. 192^8^, Fig. 22^30^), in which cladopyxidoid dinophytes are not part of the Gonyaulacales. In any case, the inferred phylogenetic position of *F. setacea*, gen. & sp. nov*.*, challenges the assumption that a partiform hypotheca would be a distinctive configuration of gonyaulacoid dinophytes^8,18^. Thus, contacts between plates Sp and 1′′′ (Z and Iu in Taylor-Evitt notation), as well as between plates 2′′′′ and 6′′′ (Y and VI in Taylor-Evitt notation), do not represent a rare, specialized gonyaulacoid plate pattern, but correspond to the common and regular configuration of peridinioid dinophytes^31^. It should be noted that while the peridinioids display only five postcingular plates, this is probably a derived character state. Furthermore, this combination of plate contacts and six postcingular plates has also been found in thecate suessialean dinophytes^43–45^.

Similar conclusions about homologies in the hypothecal plate pattern have already been drawn for Amphidomataceae^24,46^, which were, like Cladopyxidaceae, formerly included within Gonyaulacales^8,14,33^. Dinophytes with a partiform hypotheca exhibit a combination of ancestral conditions, such as six postcingular plates (traditionally associated with Gonyaulacales) and the symmetrical arrangement of plates (traditionally associated with Peridiniales). Plesiomorphic traits are unsuitable to support close relationships and thus, the assemblage of dinophytes with a partiform hypotheca does not represent a monophylum in the DNA tree.

**Figure 9:**
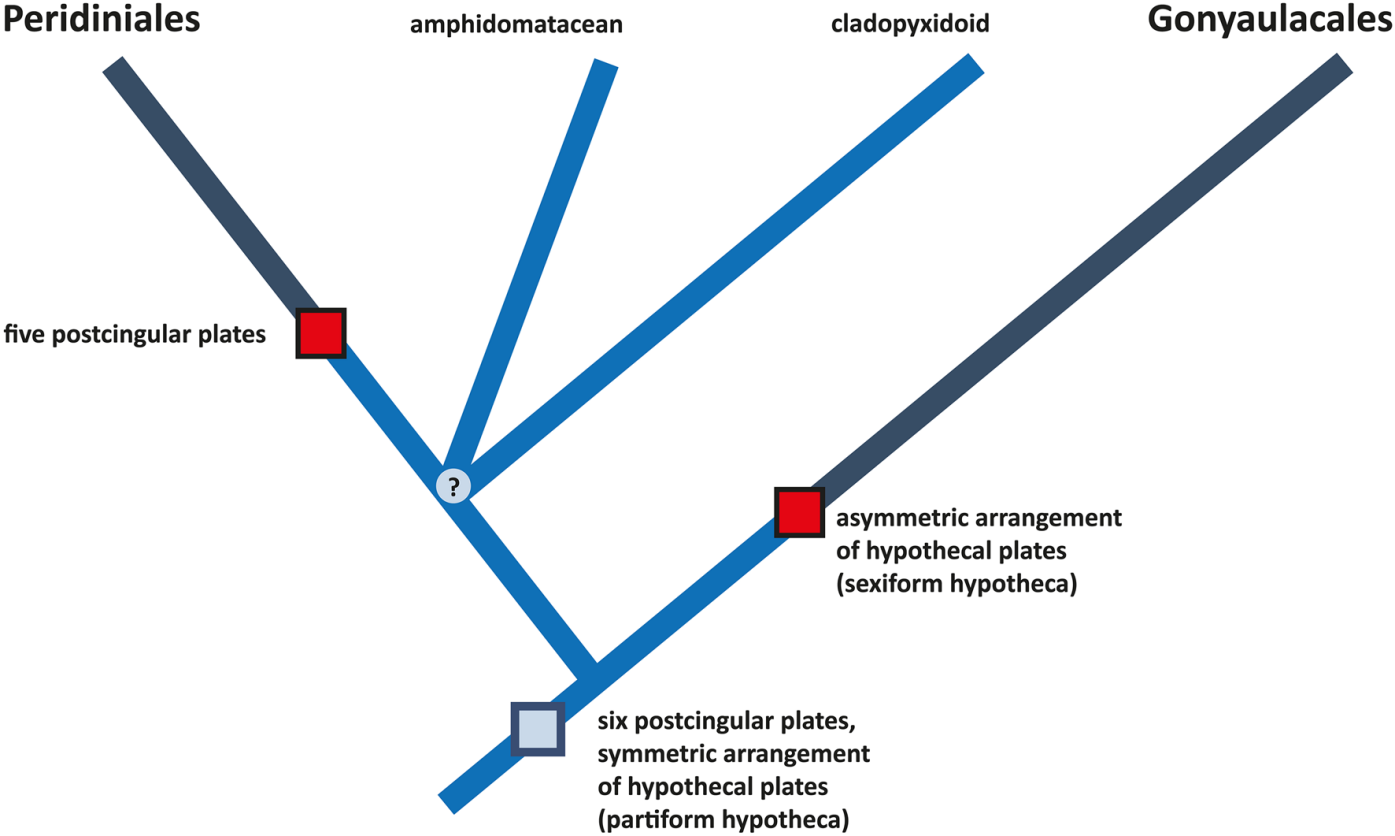
Summary cladogram of thecate dinophytes, excluding specialized forms such as dinophysalean, prorocentralean and suessialean dinophytes, with apomorphies indicated as red boxes and ancestral state as a grey box. Note that only the ancestral sexiform stage is considered here from which the corniform and quinqueform states derived within Gonyaulacales^21^.

Character evolution of thecate dinophytes may take place (in a top-down approach, not considering fossils) from ancestral conditions towards thecal patterns with only five postcingular plates (in Peridiniales) and more asymmetrical arrangements (in Gonyaulacales) as derived character states, respectively (Fig. 9). The reduction of postcingular plates in Peridiniales (probably a fusion of plates 3′′′ and 4′′′ that are still present in extant clado-pyxidoid dinophytes; Fig. 1) is most likely an independent evolution of some gonyaulacalean dinophytes^47–49^, in which size reduction of the proximate postcingular plate may also lead to fewer elements in this series. In any case, there is no such thing as a newly created posterior intercalary plate in Gonyaulacales, but there are rather two antapical plates (one of which is shifted towards the ventral side^50^) that appear homologous to those of the Peridiniales^23^ (Fig. 1B). The continuous but incorrect systematic placement of dinophytes exhibiting the partiform hypotheca into the Gonyaulacales may have prevented an easier but even more parsimonious interpretation of the data.

### Epithecal configurations

If a symmetrical hypotheca configuration is an ancestral character state in thecate dinophytes, then the question arises whether the epitheca also displays ancestral characters. The almost symmetrical epithecal plate pattern of *Acanthodinium*^42^ and *C. brachiolata*^12^ that have three anterior intercalary plates is reminiscent of the Peridiniales and likely plesiomorphic. *Cladopyxis hemibrachiata*^12^ shares an asymmetric epithecal plate pattern and four anterior epithecal plates with *F. setacea*, gen. & sp. nov., which are probably derived states, but the species differ in their thecal processes (see “Diversity of thecal processes”).

In the APC, *F. setacea*, gen. & sp. nov*.*, only has a pore platelet and no canal plate X, whereas other dinophytes with a partiform hypotheca, such as *Amphidoma* F.Stein and *Azadinium* Elbr. & Tillmann, show the peridinioid configuration with both plates present^24^. One of the most intriguing traits is the presence of anterior intercalary plates in many Peridiniales that are notably rare in Gonyaulacales; if present, they are in unusual dinophytes, such as *Pyrophacus* F.Stein, or in early lineages, such as †Lingulodiniaceae^21^. *Fensomea setacea*, gen. & sp. nov., also has intercalary plates, which is reminiscent of the Peridiniales. The topology may provide evidence of homology between the intercalary plates of *F. setacea*, gen. & sp. nov*.*, Amphidomataceae, and Peridiniales, but whether they correspond to small and rare plates in Gonyaulacales^8,21^ is an area for future research.

Fossils assigned to Cladopyxidaceae are found in Early Jurassic through Palaeocene marine strata^8,16,30^ and had the highest diversity in the Mesozoic. Notably, no representative fossils are known from the Quaternary, including modern deposits^14^. The asymmetric plate pattern equivalent of the late Palaeocene †*Cladopyxidium saeptum* (P.Morgenr.) Stover & Evitt (= †*Cladopyxidium septatum* D.M.McLean) is very similar to *F. setacea*, gen. & sp. nov*.* However, it is considered to represent coccoid (not thecate or flagellated) cells^51^. †*Cladopyxidium saeptum* has elevated crests, an archaeopyle, and is presumably composed of dinosporin; all of this is not known from *F. setacea*, gen. & sp. nov., as studied here. *Fensomea setacea*, gen. & sp. nov., is also similar to the Toarcian †*Cladopyxidium svalbardense* (Below) Lentin & G.L.Williams; however, this Jurassic species shows contacts between plates 3′ and 1a as well as between plates 3a and 4″^30^ (and not the contacts between plates 2′ and 2a as well as 2a and 5″ of *F. setacea*, gen. & sp. nov.). There is also similarity between *F. setacea*, gen. & sp. nov., and the Maastrichtian †*Cladopyxidium marheineckei* G.L.Williams, Lentin & Fensome, but the latter has four apical plates^52^, which has not yet been documented in extant cladopyxidoid dinophytes^12^. More research is necessary to disentangle the diverse biology and complex taxonomy of both extant and fossil cladopyxidoid dinophytes.

### Taxonomic activity

We cannot discount that the original drawings^10^ (Fig. S1) represent more than a single species, and F. von Stein himself tentatively associated his new species *C. brachiolata* with *Xanthidium furcatum* Ehrenb. and †*Xanthidium ramosum* Ehrenb., respectively. The first (non-fossil) name is accepted today for a desmidiacean green algae, namely *Staurastrum furcatum* (Ehrenb.) Bréb., to which Christian G. Ehrenberg erroneously assigned also Cretaceous fossils^53^ (the misinterpretation of the name was sustained over a long period of time^54^). However, Figs. 12 and 13^10^ may in fact represent the gonyaulacacean †*Spiniferites mirabilis* (M. Rossignol) Sarjeant or a similar species.

Below, we lectotypify *C. brachiolata* based on one of the original illustrations. The cell selected for this taxonomic act corresponds to the most widely applied concept of the species^8,12,33^. The lectotype designated here should be substantiated by epitypification based on newly collected material and investigated with contemporary methods, such as electron microscopy and molecular sequence diagnostics, to ensure unambiguity of the name’s application.

It is noteworthy that F. von Stein found algal remnants assigned to *Cladopyxis* in the digestive tracts of thaliacean tunicates from the Atlantic Ocean and the South Seas. Additionally, F. von Stein considered *Cladopyxis* (and other dinophytes such as *Heterocapsa* F.Stein^55^) to be an animal, so its publication falls under the rules of the *International Code of Zoological Nomenclature*^56^. No restrictions are conceivable in this case, and the name is, therefore, validly published under Art. 45.1 of the *International Code of Nomenclature for algae, fungi, and plants (Shenzhen Code)*^57^.

### Cladopyxis brachiolata

F.Stein, Der Organismus der Flagellaten nach eigenen Forschungen in systematischer Reihenfolge bearbeitet 3.2: 19, pl. II 7–13. 1883. *Xanthidium brachiolatum* (F.Stein) K.Möbius, Wissenschaftliche Meeresuntersuchungen 12: 124, pl. VIII 60–61. 1887.—Type: without precise locality (Atlantic Ocean, the South Seas), without date [non-fossil protists found in Thaliacea (Urochordata)]: *Anonymous s.n.*—**Lectotype** (**designated here**): [illustration] Der Organismus der Flagellaten nach eigenen Forschungen in systematischer Reihenfolge bearbeitet 3.2: pl. II 7.—Other original elements may comprise other species of dinophytes (pl. II 9: *Cladopyxis quadrispina* Pavill., pl. II 11: *Cladopyxis steinii* O.Zacharias) and/or protists from other organismal lineages (e.g., pl. II 12–13, see above) and are to be disregarded (ICN Arts 8.2, 9.14). Figure S1. This taxonomic act has been registered in PhycoBank under http://phycobank.org/102641.

### Fensomea setacea Tillmann & Gottschling, gen. & sp. nov.

—Type [SEM-stub with non-fossil specimens prepared from material fixed with formaldehyde]: western South Atlantic (31°25'S, 37°31'W), 7 Mar 2000: [Meteor 46/4] [M. Kirsch GeoB*184] 3/7/a (**holotype**, **designated here**: CEDiT-2020H121!, **isotype**, **designated here**: CEDiT-2020I122!). Figures 2–6. This taxonomic act has been registered in PhycoBank under http://phycobank.org/102642.

Description (ICN Art. 38.5: descriptio generico-specifica): Dinophytes small, phototrophic, thecate, thecal plate pattern distinct. Cells 14–23 µm long, 14–21 µm wide, spherical to oval in outline, the surface echinate through baculate, with scattered setae ranging 2–6 µm. Tabulation formula: APC, 3′, 4a, 7″, 7C, 6S, 6′′′, 2′′′′; plates Sp and 1′′′ adjacent, plates Sp and 5′′′ separated; epithecal keystone plate 3″, hypothecal keystone plate 4′′′, plate 2a overlapped by all adjacent plates. No ventral pore present. Coccoid cells unknown.

Notes: Delimitation from other cladopyxidoid dinophytes (i.e., a diagnosis) is provided in the “Discussion” (particularly “Diversity of thecal processes”). We consider the slender processes (here referred to as setae) to be the most striking diagnostic trait to delimit our new taxon from previously described ones. The generic name honours Robert A. Fensome, who contributed enormously to the knowledge of extant and fossil dinophytes and who accentuated the phylogenetic importance of cladopyxidoid dinophytes as a link between the Gonyaulacales and the Peridiniales^15^.

## Methods

### Collection, strain establishment, and morphology

Strain GeoB*184 was established from a single cell recovered from material from the South Atlantic (R/V *Meteor* cruise 46/4^58^; wheel pump 3/7/a; ca 31° 25′ S, 37° 31′ W; 23 °C surface temperature; salinity: 35.5) collected on 7 March 2000. The phototrophic strain was maintained in a Percival I-36VL climate chamber (CLF Plant-Climatics; Emersacker, Germany) at 23 °C, 80 µmol photons m^−2^ s^−1^, and a 12:12 h light:dark photoperiod, using K-Medium without silicate^59^ in 35 psu artificial seawater at pH 8.0–8.2^60^. The strain decayed several months after its establishment so living material could not be inspected; unfortunately, this decay occurred before its taxonomic features were fully clarified. Therefore, we studied material, using our standard laboratory procedures, that had previously been fixed (almost 20 years before) with formaldehyde (2% final concentration).

Our LM work used an Axioskop 2 microscope (Zeiss, Göttingen, Germany) with differential interference contrast (DIC) and 1000× magnification. Cells were documented with a digital camera (MRC5, Zeiss). For SEM observations, cells were collected on polycarbonate filters (Millipore, 25 mm diameter, 3 µm pore size) in a filter funnel, in which all subsequent washing and dehydration steps were carried out. Eight washing steps (2 ml of MilliQ-deionized water each) were followed by a series of dehydration steps in ethanol (30%, 50%, 70%, 80%, 95%, and 100% at 10 min for each step). Filters were dehydrated with hexamethyldisilazane (HMDS) in 1:1 HMDS:EtOH and then twice in 100% HMDS and stored in a desiccator under a vacuum. Finally, the filters were mounted on stubs, sputter-coated (SC500, Emscope, Ashford, UK) with gold–palladium and viewed with a Quanta FEG 200 SEM (FEI, Eindhoven, Netherlands). Some SEM micrographs were presented on a black background using Adobe Photoshop 6.0 (Adobe Systems, San Jose, California, USA). The labelling of dinophyte thecal plates was performed according to the Kofoidean system^22,23^.

### Molecular phylogenetics

For DNA isolation, fresh material was processed using the the Nucleo Spin Plant II Kit (Machery-Nagel; Düren, Germany). Various loci of the rRNA operon (i.e., SSU, ITS, LSU) were amplified using primer pairs specified previously and following standard protocols^18,49^. Gel electrophoresis yielded single bands that were purified. PCR products were sequenced directly in both directions using the ABI Big-Dye dye-terminator technique (Applied Biosystems; Foster City, USA‒CA), according to the manufacturer’s recommendations, and a ABI 3730 capillary sequencer (Applied Biosystems). Sequences were edited and assembled using Sequencher™v5.1 (Gene Codes; Ann Arbor, USA‒MI). For visual comparison of the edited sequences, the alignment editor ‘Se-Al’ (http://tree.bio.ed.ac.uk/software/seal/) was used.

To compute a dinophyte reference tree inferred from a concatenated rRNA alignment^29,61^, we compiled a systematically representative set comprising 152 dinophytes (plus nine outgroup accessions; Table S1). For alignment constitution, separate matrices of the rRNA operon were constructed, aligned using ‘MAFFT’ v6.502a^62^, and the −qinsi option to consider the secondary structure, and concatenated afterwards. The aligned matrices are available as Fensomea.nex file in the supplement.

Phylogenetic analyses were carried out using Maximum Likelihood (ML) and Bayesian approaches, as described^63^, using the resources available from the CIPRES Science Gateway^64^. Briefly, the Bayesian analysis was performed using ‘MrBayes’ v3.2.7a^65^ (freely available at http://mrbayes.sourceforge.net/download.php) under the GTR + Γ substitution model and the random-addition-sequence method with 10 replicates. We ran two independent analyses of four chains (one cold and three heated) with 20,000,000 generations, sampled every 1000th cycle, with an appropriate burn-in (10%) inferred from evaluation of the trace files using Tracer v1.7.1^66^. For the ML calculations, the MPI version of ‘RAxML’ v8.2.4^67^ (freely available at http://www.exelixis-lab.org/) was applied using the GTR + Γ substitution model under the CAT approximation. We determined the best-scoring ML tree and performed 1000 non-parametric bootstrap replicates (rapid analysis) in a single step. Statistical support values (LBS: ML bootstrap support; BPP: Bayesian posterior probabilities) were drawn on the resulting, best-scoring tree.

## Supplementary Information

Supplementary Information 1.

Supplementary Information 2.
