# Supplementary material for: Fensomea setacea, gen. & sp. nov. (Cladopyxidaceae, Dinophyceae), is neither gonyaulacoid nor peridinioid as inferred from morphological and molecular data
Source: Sci Rep. 2021 Jun 17;11:12824. doi: 10.1038/s41598-021-92107-0 (PMC8211658; doi:10.1038/s41598-021-92107-0)
Supplement: Supplementary file 1 — Supplementary Information 1. [file 41598_2021_92107_MOESM1_ESM.pdf]

Fensomea setacea, gen. & sp. nov. (Cladopyxidaceae, Dinophyceae), is neither gonyaulacoid nor peridinioid as inferred from morphological and molecular data

Marc Gottschling, Maria Consuelo Carbonell-Moore, Kenneth Neil Mertens, Monika Kirsch, Malte Elbrächter, Urban Tillmann

Supplementary Material

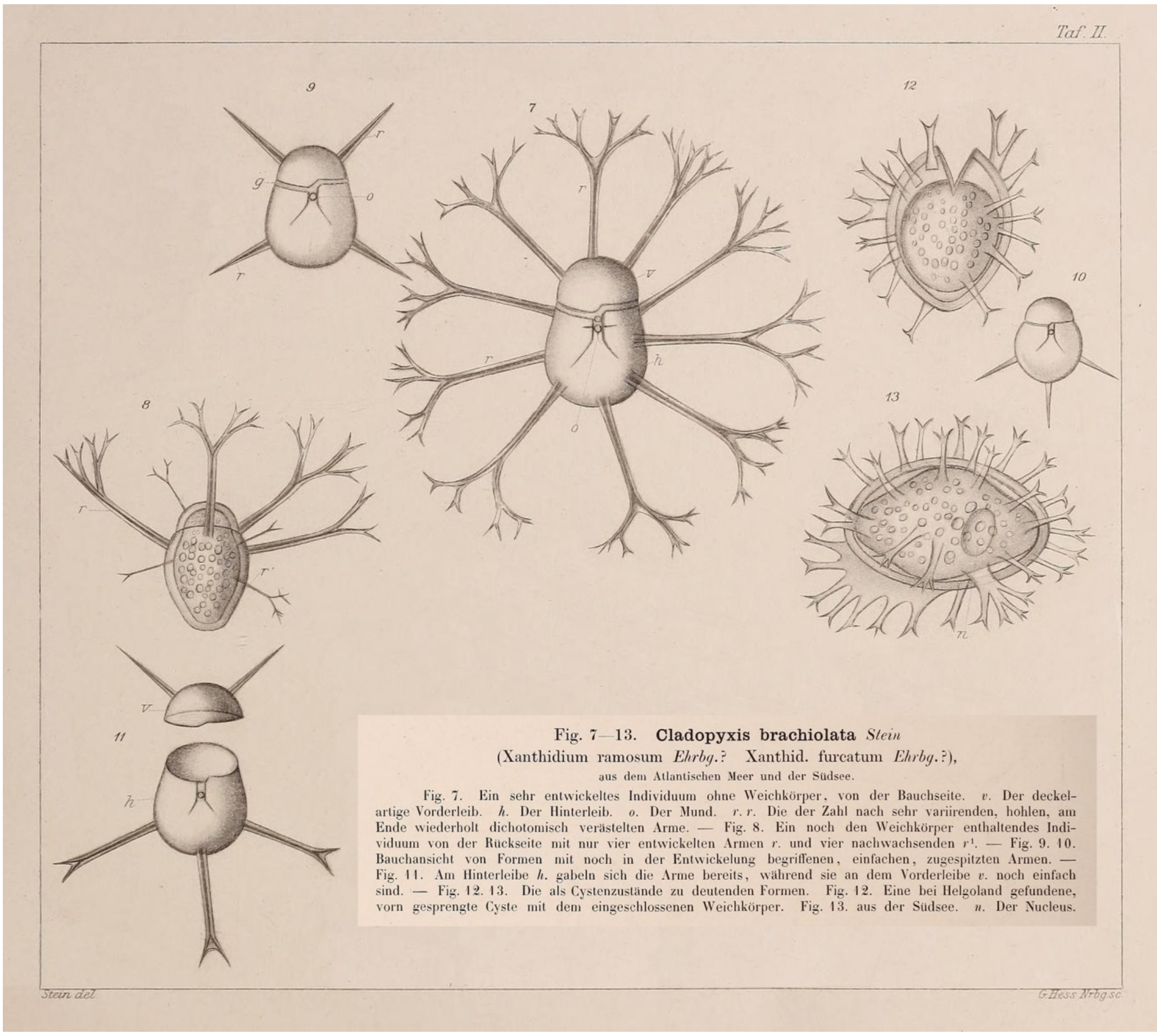

Figure S1: Friedrich v. Stein's original material of *Cladopyxis brachiolata*
(reproduction of pl. II 7–13). Note that pl. II 7 is chosen as lectotype. Furthermore, pl. II 13 is likely gonyaulacean †*Spiniferites mirabilis* (M.Rossignol) Matsuoka and pl. II 12 possibly also a species of †*Spiniferites* Mantell.

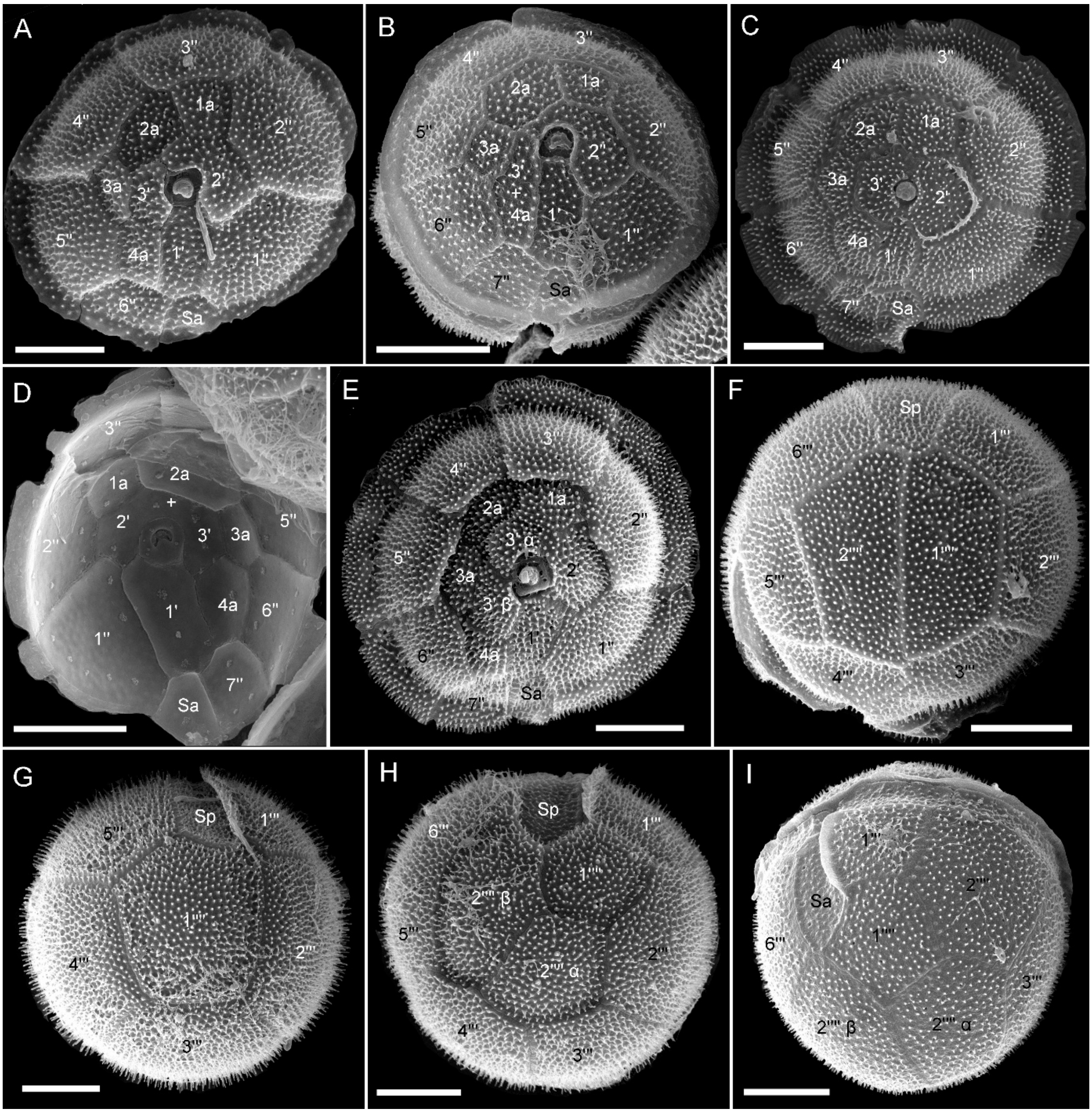

Figure S2: Variability in plate pattern and arrangement of *Fensomea setacea*, gen & sp. nov., (GeoB*184) regarding (A–E) epithecal plates and (F–I) hypothecal plates. (A) Presence of only six precingular plates. (B) Loss of one epithecal plate interpreted here as a fusion of plates 3' and 4a. (C) Basic plate pattern, but unusually plates 2' and 3' are of equal size and in lateral position. (D) Loss of one epithecal plate interpreted here as fusion of apical plates 2' and 3'. (E) Presence of one additional epithecal plate interpreted here as a subdivison of plate 3'. (F) Basic plate pattern, but unusually plates 1'''' and 2'''' are of equal size and symmetrically arranged. Note that the list of plate 1''' towards plate Sp is missing. (G) Loss of two hypothecal plates, only 5 precingular plates and one antapical plate present. (H–I) Presence of three antapical plates, interpreted here as a subdivision of plate 2''''. Scale bars = 5 µm.

Tables

Table S1: Voucher list. All names are given under the rules of the ICN, the author standard forms follow (Brummitt and Powell 1992). Abbreviation: n. inf., no information. If 'holotype' or 'epitype' is noted for a species name, then it refers to material, from which the type was prepared.

Species name with author	Strain No.	Locality	Date	Collector(s)	GenBankNo(s)	Reference
outgroup						
Amoebophrya sp. [isolated from *Levanderina fissa* (Levander) Moestrup, Hakanen, Gert Hansen, Daugbjerg & M.Ellegaard]	n.inf.	western North Atlantic, off USA–MD: Chesapeake Bay	n.inf.	n.inf.	HM483394 (SSU+ITS+LSU)	Coats et al. (2010)
Amoebophrya sp. [isolated from *Akashiwo sanguinea* (K.Hirasaka) Gert Hansen & Moestrup]	n.inf.	western North Atlantic, off USA–MD: Chesapeake Bay	n.inf.	n.inf.	HM483395 (SSU+ITS+LSU)	Coats et al. (2010)
Besnoitia besnoiti (E.E.Franco & I.Borges, 1916)		Israel	n.inf.	n.inf.	DQ227420 (rRNA)	Cortes et al. (unpubl. 2005)
Euduboscquella sp. [isolated from *Favella arcuata* (K.Brandt, 1906)]	OC20	USA–MD: Ocean City, Assawoman Bay (38°20'N, 75°06W)	Aug 11, 2010	n.inf.	JN934989 (SSU+ITS+LSU)	Bachvaroff et al. (2012)
marine alveolate	FBB25	USA–MA: Boston, Blanes Bay Microbial Observatory	n.inf.	n.inf.	EU304548 (rRNA)	Massana et al. (2008)

Perkinsus andrewsi Coss, J.Robledo, G.Ruiz & Vasta, 2001 [isolated from *Macoma balthica* (Linnaeus, 1758)]	ATCC50807 (≡ PAND-A8–4a)	USA–MD: Rhode River	n.inf.	n.inf.	AY305326 (rRNA)	Pecher et al. (2004)
Perkinsus atlanticus C.Azevedo, 1989 (isolated from *Venerupis decussata* Linnaeus, 1758)	ALG1	Portugal	n.inf.	n.inf.	AF509333 (rRNA)	Robledo et al. (2002)
Toxoplasma gondii (Nicolle & Manceaux, 1908) (isolated from *Homo sapiens* Linnaeus, 1758)	P	n.inf.	n.inf.	n.inf.	X75453 (SSU+ITS+LSU)	Ding et al. (unpubl. 1993)
Vitrella brassicaformis Oborník, D.Modrý, M.Lukeš, Cernotíková-Stříbrná, Cihlář, Tesařová, Kotabová, Vancová, Prášil & J.Lukeš (isolated from an anthozoan species)	NCMA3155 (≡ RM11)	off Australia: Great Barrier Reef (23°30'S, 152°00'E)	Jan 12, 2001	K. Miller & C. Mundy [R.A. Andersen & R. Moore] s.n.	HM245049 (rRNA)	Janouškovec et al. (2010)
<u>unplaced dinophytes</u>						
Akashiwo sanguinea (K.Hirasaka) Gert Hansen & Moestrup	GnSg02	western North Pacific, East China Sea, off South Korea: Jangmok	n.inf.	M. Chang s.n.	AY831410 (rRNA)	Ki and Han (2005)
Akashiwo sanguinea (K.Hirasaka) Gert Hansen & Moestrup	NCMA1837 (≡ CCCM885)	western North Atlantic, Sargasso Sea, off UK, the Bermudas: Harington Sound off Rabbitt Island (32°20'N, 64°44'W)	Sep 23, 1997	S. Sarkis s.n.	DQ779988 (rRNA)	Ki and Han (2007a)

Amphidinium carterae Hulburt	NCMA124 (≡ CCCM439, UW380)	eastern North Pacific, off Mexico: Sonora, Puerto Penasco (31°19'N, 113°34'W)	Jun 20, 1966	R. Norris s.n.	AF274255 (SSU), EU927575 (ITS), AY460584 (LSU)	Saldarriaga Echavarría et al. (2001), Murray et al. (2004) Ferrell & Beaton (unpubl. 2008)
Amphidoma languida Tillmann, R.Salas & Elbr.	2A11	North Atlantic, off Iceland (65°27'N, 24°39'W)	Aug, 2012	U. Tillmann [Maria S. Merian] [U. Tillmann] s.n.	KR362880 (SSU), KR362882 (ITS), KR362885 (LSU)	Tillmann et al. (2015)
Ankistrodinium armigerum K.Watanabe, Miyoshi, F.Kubo, Sh.Murray & T.Horig.	n.inf.	western North Pacific, Sea of Japan, off Japan: Hokkaidō, Ishikari Beach (43°15'N, 141°21'E)	n.inf.	n.inf.	AB858349 (SSU), AB858350 (LSU)	Watanabe et al. (2014)
Apicoporus glaber (Hoppenrath & Okolodkov) Sparmann, B.S.Leander & Hoppenrath	n.inf. (chimeric)	Germany: Sylt	Mar, 2009	n.inf.	EU293235 (SSU), JQ179867 (LSU)	Sparmann et al. (2008), Hoppenrath et al. (2012)
Azadinium caudatum var. caudatum (Halldal) Nézan et Chomérat	IFR1191 [IFR10-332, IFR10-330, IFR11-033]	eastern North Atlantic, Celtic Sea, off France: Brittany, Finistère, Concarneau (47°50'N, 3°57'W)	Dec 15, 2009	[E. Nézan] s.n.	JQ247701 (SSU), JQ247700 (ITS), JQ247702 (LSU)	Nézan et al. (2012)
Azadinium concinnum Tillmann & Nézan (holotype)	1C6 (single and multiple cells, LSU clone 4)	North Atlantic, Irminger Sea, off Greenland (62°14'N, 37°27'W)	Aug, 2012	U. Tillmann [Maria S. Merian] [U. Tillmann] s.n.	KJ481826 (SSU), KJ481827 (ITS), KJ481831 (LSU)	Tillmann et al. (2014)
Azadinium poporum Tillmann & Elbr.	TIO256	Mediterranean, off France: Corsica (42°08'N, 9°32'E)	Jan 18, 2016	n.inf.	MF033112 (SSU), MF033116 (ITS), MF033123 (LSU)	Luo et al. (2017)
Azadinium spinosum Elbr. & Tillmann	SHETF6	North Sea, off Shetland Islands (60°13'N, 1°00'W)	May, 2011	U. Tillmann [Heincke] [U. Tillmann] s.n.	JX559885 (SSU+ITS+LSU)	Tillmann et al. (2012)

Bernardinium sp.	CCP2	USA–NC: Pond near Cashiers (35°08'N, 83°05'W)	Jul, 2009	n.inf.	JQ439940 (rRNA)	Fawcett and Parrow (2012)
Bispinodinium angelaceum N.Yam. & T.Horig. (holotype)	HG236	western North Pacific, off Japan: Kyūshū, Kagoshima, off Mageshima (30°41' N, 130°50'E)	May 15, 2008	R. Terada s.n.	AB762397 (SSU), AB762398 (LSU)	Yamada et al. (2013)
Brachidiniaceae sp.	GrAr01	western North Pacific, Sea of Japan, off South Korea: Chilchondo	n.inf.	n.inf.	DQ779991 (rRNA)	Ki and Han (2007a)
Bysmatrum subsalsum (Ostenf.) M.A.Faust & Steid.	KC32CCAUTH	off Greece, North Aegean Sea: Thessaloniki, Porto-Lagos (40°58'N, 25°07'E)	n.nf.	N. Nikolaidis s.n.	HQ845326 (SSU+ITS+LSU)	Gottschling et al. (2012)
Ceratoperidinium falcatum (Kof. & Swezy) A.Reñé & de Salas	IFR1100	eastern North Atlantic, Celtic Sea, off France: Brittany, Finistère, Douarnenez (48°10'N, 4°24'W)	Oct 5, 2009	REPHY	KJ508394 (LSU)	Nézan et al. (2014)
Fensomea setacea Tillmann & Gottschling (holotype)	GeoB*184	western South Atlantic (31°25'S, 37°31'W, –5m)	Mar 7, 2000	K.J.S. Meier & M. Streng [Meteor 46/4] [M. Kirsch] WP 3/7/a	MW267274 (SSU+ITS+LSU), MW267282 (LSUd8d10)	this study
Gertia stigmatica K.Takahashi, G.Benico, Wai Mun Lum & M.Iwataki	mdd472-kt	western North Pacific, off Japan, Sagami Bay: Honshū, Kantō, Kanagawa, Manazuru (35°09'N, 139°10'E)	Oct 20, 2016	K. Takahashi s.n.	LC490696 (rRNA)	Takahashi et al. (in press)

Glenodiniopsis steinii Wołosz. (reference material)	NIES463 (≡ TM3D6)	Japan: Iwate, Shizukuishi	Sep 10, 1984	[T. Sawaguchi] s.n.	AF274257 (SSU), EF058255 (LSU)	Saldarriaga Echavarría et al. (2001), Logares et al. (2007b)
"*Gymnodinium*" *impatiens* Skuja	CCAC0025 (≡ M0925)	Germany: Brandenburg, Neuglobsow	1992	[B. Marin] s.n.	EF058239 (SSU), EF058259 (LSU)	Logares et al. (2007b)
Gyrodinium dominans Hulburt	GDMS0704YD	western North Pacific, East China Sea, off South Korea: Gyeongsangnam-do, Masan Bay	Apr 1, 2007	Y.D. Yoo s.n.	FN669510 (SSU+ITS+LSU)	Yoon et al. (2012)
Gyrodinium helveticum (Penard) Y.Takano & T.Horig. (reference material)	K1	Russia, Lake Baikal: Irkutsk, between Listvyanka and Bolshie Koty	2012	n.inf.	MG493227 (SSU), MG255302 (ITS+LSU)	Annenkova (2018)
Hemidinium nasutum F.Stein (reference material)	NIES471 (≡ 87SPD1)	Japan: Honshū, Ibaraki, Tsuchiura	Aug 27, 1987	[T. Sawaguchi s.n.]	AY443016 (SSU), EF058260 (LSU)	Saldarriaga Echavarría et al. (2004), Logares et al. (2007b)
Jadwigia applanata Moestrup, K.Lindb. & Daugbjerg (holotype)	CCAC0021 (≡ SCCAP K-1115)	Germany: Hessen, Biebergemünd, near Lochmühle	1991	[A. Schilke] s.n.	EF058240 (SSU), AY950447 (LSU)	Lindberg et al. (2005), Logares et al. (2007b)
Karenia mikimotoi (Miyake & Komin. ex M.Oda) Gert Hansen & Moestrup	NCMA429 (≡ CCAP1127/1, G1, PLY497a)	UK: England, Devon, Plymouth, Sutton Harbour (50°22'N, 4°10'W)	Aug 23, 1980	D. Harbour s.n.	FJ587220 (SSU), HM807318 (ITS), AF200678 (LSU)	Hansen et al. (2000), Garces et al. (2006), Hou et al. (unpubl. 2008), Stern et al. (2010), Al-Kandari et al. (2011)
Kirithra asteri Boutrup, Tillmann, Daugbjerg & Moestrup	H-1-A6	western South Atlantic, off Argentina (41°11'S, 57°52'W, –5m)	Sep 9, 2015	U. Tillmann [Houssay] [U. Tillmann] s.n.	MW267281 (SSU), MW267275 (ITS), MF666674 (LSU)	Boutrup et al. (2017), this study

Levanderina fissa (Levander) Moestrup, Hakanen, Gert Hansen, Daugbjerg & M.Ellegaard	NCMA431 (≡ CCCM796, LISBOA176)	Portugal: near Santiago do Cacem, Santo Andre lagoon (38°03'N, 8°48'W)	Nov 1, 1980	E. Silva s.n.	AY443015 (SSU), JQ972685 (ITS), EF205007 (LSU)	Saldarriaga Echavarría et al. (2004), Moestrup and Daugbjerg (2007), Stern et al. (2012)
Moestrupia sp.	AW22-12	western North Pacific, off Japan: Okinawa, Awase (26°19'N, 127°50'E)	Apr 22, 2013	S. Suda s.n.	LC025890 (SSU), LC025909 (ITS), LC025928 (LSU)	Prabowo et al. (2016)
Moestrupia sp.	AW22-15	western North Pacific, off Japan: Okinawa, Awase (26°19'N, 127°50'E)	Apr 22, 2013	S. Suda s.n.	LC025892 (SSU), LC025911 (ITS), LC025930 (LSU)	Prabowo et al. (2016)
Moestrupia sp.	G4M4	western North Pacific, East China Sea, off Japan: Okinawa, Onna, Maeda (26°26'N, 127°46'E)	Apr 3, 2013	S. Suda s.n.	LC025882 (SSU), LC025901 (ITS), LC025920 (LSU)	Prabowo et al. (2016)
Noctiluca scintillans (Macartney) Kof. & Swezy	n.inf.	off China: Hong Kong, Clear Water Bay (22°20'N, 114°16'E)	Mar 26, 2006	J.-S. Ki s.n.	GQ380592 (SSU+ITS+LSU)	Ki (2010)
Nottbeckia ochracea (Levander) Gert Hansen, Daugbjerg & Moestrup (reference material)	GH957	Finland: Uusimaa, Raseborg, Tvärminne, Brännskär	Jun 19, 2011	n.inf.	MG754078 (SSU), MG754079 (LSU)	Hansen et al. (2018)
Peridiniella catenata (Levander) Balech	SCCAP K-0543	n.inf.	n.inf.	n.inf.	AF260398 (LSU)	Daugbjerg et al. (2000)
Peridiniella sp.	NC-2011	eastern West Atlantic, off France: Brittany, Finistère, Brest	Jun 7, 2010	n.inf.	### (SSU), JQ247714 (LSU)	Nézan et al. (2012)
Pseliodinium pirum (F.Schütt) F.Gómez	PHJZB1	western North Pacific, Yellow Sea, off China, Jiaozhou Bay: Qingdao (36°06'N, 120°15'E)	Aug 11, 2015	n.inf.	MH469533 (SSU), MH469535 (LSU)	Hu et al. (2020)

Takayama acrotrocha (J.Larsen) de Salas, Bolch & Hallegr.	MC728-D5	Mediterranean Sea, Tyrrhenian Sea, off Italy: Campania, Naples	Aug 22, 2006	n.inf.	HM067010 (SSU), HM067011 (ITS), FJ024703 (LSU)	Henrichs et al. (2011), Siano et al. (2009)
Togula jolla M.F.Jørg., Sh.Murray & Daugbjerg (holotype)	UTEX1562	eastern North Pacific, off USA–CA: San Diego, La Jolla	n.inf.	A.R. Loeblich s.n.	AF274252 (SSU), AY455680 (LSU), AF482405 (tub)	Saldarriaga Echavarría et al. (2001, 2003), Jørgensen et al. (2004)
Tovellia cf. *aveirensis* Pandeir., Craveiro, Daugbjerg, Moestrup & Calado	TSJL01	China: Fujian, Jiulong Jiang	Apr 28, 2013	n.inf.	KU359052 (SSU), KU359051 (ITS), KU359050 (LSU)	Luo et al. (2016)
Tovellia sp.	HBI:SC201101a	n.inf.	n.inf.	n.inf.	JQ639766 (SSU), JQ639774 (ITS), JQ639756 (LSU)	Zhang et al. (unpubl. 2012)
<u>Gymnodiniales</u>						
Barrufeta resplendens (Hulburt) H.Gu, Z.Luo & K.N.Mert.	GM17	western North Atlantic, Gulf of Mexico, off USA–LA (28°52'N, 90°29'W)	Jul 29, 2014	n.inf.	KY688183 (SSU), KT203384 (ITS), KT203382 (LSU)	Gu et al. (2015), Wang et al. (2017)
Chytriodinium sp. (isolated from a copepod egg sac)	Atlantic isolate 7	South Atlantic, off Brazil: São Paulo, Sao Sebastiao Channel (23°50'S, 45°24'W)	Apr 30, 2013	n.inf.	KM245128 (SSU+ITS+LSU)	Gómez and Skovgaard (2014)
Gymnodinium aureolum (Hulburt) Gert Hansen	GeoB 232	Mediterranean Sea, Ionian Sea, off Italy: Gulf of Taranto (40°07'N, 17°19'E)	Oct 26, 2002	D. Saracino [M. Kirsch] s.n.	KJ481834 (SSU+ITS+LSU)	Tillmann et al. (2014)
"*Gymnodinium*" *catenatum* H.W.Graham	GnCt01	eastern Indian Ocean, East China See, off South Korea: Nanpo, Jinhae Bay	n.inf.	n.inf.	DQ785882 (rRNA)	Ki and Han (2007b)

Gymnodinium fuscum var. *rubrum* Baumeister ex Romeikat, Knechtel & Gottschling (epitype)	GeoM*864 (≡ CCAC9044B)	Germany: Bavaria, Traunstein, Seeon, peat pit (47°59'N, 12°26'E, 536m)	Jun 28, 2017	C. Romeikat, M. Gottschling & H. Reich [C. Romeikat] D099	MK405489 (SSU+ITS+LSU)	Romeikat et al. (2020)
Gymnodinium plasticum Na Wang, Z.Luo, K.N.Mert., F.M.G.McCarthy & H.Gu (holotype)	TIO826	Canada: Ontario, Plastic Lake (45°18'N, 79°23'E)	n.inf.	n.inf.	KY688188 (SSU), KY688186 (ITS), KY688184 (LSU)	Wang et al. (2017)
Gymnoxanthella sp. (isolated from *Spongotrochus glacialis* Popofsky, 1908)	n.inf.	North Pacific	fall, 2010	n.inf.	AB860180 (rRNA)	Ishitani et al. (2014)
Gyrodiniellum shiwhaense N.S.Kang, H.J.Jeong & Moestrup (holotype)	n.inf.	western North Pacific, Yellow Sea, off South Korea: Shiwha Bay (37°07'N, 126°08'E)	Sep 1, 2009	N.S. Kang s.n.	FR720082 (SSU+ITS+LSU)	Kang et al. (2011)
"*Gyrodinium*" *impudicum* S.Fraga & I.Bravo	Gi-1cp	eastern Indian Ocean, East China See, off South Korea: Yeosu	n.inf.	n.inf.	DQ779992 (rRNA)	Ki and Han (2007a)
Lepidodinium viride M.Watan., S.Suda, I.Inouye, Sawaguchi & Chihara	n.inf.	South Africa	n.inf.	R.N. Pienaar s.n.	DQ499645 (SSU+ITS+LSU)	Grzebyk et al. (unpubl. 2008)
Nematodinium sp.	UBC3 (chimeric)	eastern North Pacific, off Canada: British Columbia, Vancouver Island (48°50'N, 125°08'W)	April, 2006; April 28, 2007	Anonymous BSL-2009a	FJ947038 (SSU), FJ947041 (LSU)	Hoppenrath et al. (2009)
Nusuttodinium aeruginosum (F.Stein) Y.Takano & T.Horig.	Gaer-Japan #1	Japan: Kagawa, Uto-ike Pond	Feb 9, 2001	Y. Takano s.n.	AB921311 (rRNA)	Takano et al. (2014)

Spiniferodinium limneticum (Wołosz.) Kretschmann & Gottschling	GeoM 517 (≡ CCAC5092B, CCBA AA-276)	Poland: Lesser Poland, Tatra, Zakopane (49°17'N, 19°57'E)	Sep 11, 2012	M. Gottschling, C. Zinßmeister, N.H. Filipowicz & P.M. Owsianny [J. Kretschmann P6] PL002	KR362900 (SSU+ITS+LSU)	Kretschmann et al. (2015)
Spiniferodinium palustre (A.J.Schill.) Kretschmann & Gottschling (reference material)	GeoM*719	Poland: Lesser Poland, Tatra, Litworowy Staw Gąsienicowy (49°14'N, 20°00'E, 1618m)	Sep 22, 2015	P.M. Owsianny [J. Kretschmann] PL018	MH497023 (SSU+ITS+LSU), MH497042 (LSUd8d10)	Žerdoner Čalasan et al. (2019)
Warnowia sp.	BC	eastern North Pacific, off Canada: British Columbia, Vancouver Island (48°50'N, 125°08'W)	May 2, 2007	Anonymous BSL-2009a	FJ947040 (SSU), FJ947042 (LSU)	Hoppenrath et al. (2009)
†Suessiales						
Ansanella granifera H.J.Jeong, S.H.Jang, Moestrup & N.S.Kang (holotype)	AGSW10	western North Pacific, Yellow Sea, off South Korea: (37°18'N, 126°36'E)	Sep, 2010	n.inf.	HG529978 (SSU), HG529979 (ITS), HG529980 (LSU)	Jeong et al. (2014a)
Asulcocephalium miricentonis K.Takahashi, Moestrup & M.Iwataki	mi11-8kt (≡ NIES3775)	Japan: Yamagata, Kenmin-no-Mori, Mikokubo pond (38°15'N, 140°12'E)	Jul, 2011	K. Takahashi s.n.	LC068836 (rRNA)	Takahashi et al. (2015)
Baldinia anauniensis Gert Hansen & Daugbjerg (holotype)	greenGS	Italy, Trentino, Lake Tovel	Jul 29, 2003	n.inf.	EF052682 (SSU), EF052683 (LSU)	Hansen et al. (2007)
Borghiella sp.	CCAC0075 (≡ M0698, SAG 42.80, SCCAP K-1119)	Germany: Lower Saxony, Göttingen, Botanical Garden of the university	1965	W. Koch s.n.	EF058253 (SSU), MG851594 (LSU)	Logares et al. (2007b), Moestrup et al. (2018)

Breviolum minutum (T.C.LaJeunesse, J.E. Parkinson & J.D.Reimer) J.E. Parkinson & T.C.LaJeunesse [isolated from *Orbicella faveolata* (J.Ellis & Sol., 1786)]	Mf 1.05b.01	western North Atlantic, Gulf of Mexico, off USA–FL	n.inf.	n.inf.	BASF01015284 (rRNA)	Shoguchi et al. (2013)
Cladocopium goreaui LaJeunesse & H.J.Jeong [isolated from *Discosoma sanctithomae* (Duchass. & Michelotti, 1860)] (holotype)	NCMA2466 (≡ 152)	western North Atlantic, Caribbean Sea, off Jamaica (18°00'N, 77°00'W)	Jan 1, 1980	S. Chang s.n.	EF036539 (SSU), FJ823600 (ITS), FJ939581(LSU)	Zhang et al. (2008), Stern et al. (2012), Yu et al. (unpubl. 2009)
Dactylodinium pterobelotum K.Takahashi, Moestrup & M.Iwataki (holotype)	vnd255-kt	western North Pacific, South China Sea, off Vietnam: Bạc Liêu, Nhà Mát (9°12'N, 105°45'E)	Mar 6, 2013	K. Takahashi s.n.	LC272997 (SSU+ITS+LSU)	Takahashi et al. (2017)
Durusdinium sp. (isolated from *Haliclona koremella* de Laub., 1954)	PSP1-05	western North Pacific, Mirconesia, off Palau: Carp Island	Sep, 1997	n.inf.	AB016578 (SSU), JN558081 (ITS+LSU), AJ308899 (LSU)	Carlos et al. (1999), Pochon et al. (2001, 2012)
Effremium voratum (H.J.Jeong, S.Y.Lee, N.S.Kang & LaJeunesse) LaJeunesse & H.J.Jeong (holotype)	SvFL1	western North Pacific, East China Sea, off Republic of South Korea: Jeju (33°28'N, 126°19'E)	2008	H.J. Jeong s.n.	HF568830 (SSU+ITS+LSU)	Jeong et al. (2014b)
Fugatium kawagutii LaJeunesse [isolated from *Montipora verrucosa* (Lam., 1816)] (holotype)	NCMA2468 (≡ 135)	North Pacific, off US–HI (21°15'N, 158°00'W)	n.inf.	R. York [R. York] s.n.	LK934666 (rRNA)	Stern et al. (2012), Lee et al. (unpubl. 2014)
Polarella glacialis Montresor, Procaccini & Stoecker (holotype)	NCMA1383	Southern Ocean, Ross Sea, off Antarctica: McMurdo Sound (77°50'S, 163°00'E)	Jun 1, 1991	D. Stoecker, M. Putt & T. Moisan s.n.	EF417317 (SSU), EU445333 (ITS), AY036080 (LSU)	Thomson et al. (2004), Logares et al. (2008, 2009)

Symbiodinium sp. [isolated from *Plexaura homomalla* (Esper, 1792)]	NCMA2456 (≡ 379)	western North Atlantic, Sargasso Sea, off UK, the Bermudas (32°23'N, 64°41'W)	Jul 29, 2004	R. Iglesias-P. [R. Iglesias-P.] s.n.	LK934674 (SSU+ITS+LSU)	Lee et al. (unpubl. 2014)
<u>Peridiniales</u>						
Aduncodinium glandulum (Herdman) N.S.Kang, H.J.Jeong & Moestrup	n.inf.	western North Pacific, East China Sea, off South Korea: Gyeongsangnam-do, Masan Bay (35°11'N, 128°35'E)	2013	N.S. Kang s.n.	LK934662 (SSU+ITS+LSU)	Kang et al. (2015)
Amyloodinium ocellatum (E.-M.Br.) E.-M.Br. & Hovasse	Ningde1412	China: Ningde	n.inf.	n.inf.	KU761581 (SSU+ITS+LSU)	Huang et al. (unpubl. 2016)
Amphidiniopsis uroensis Toriumi, Yoshimatsu & J.D.Dodge	uro8	western North Pacific, off Japan: Kōchi (33°19'N, 134°10'E)	Apr 18, 2014	A. Yamaguchi s.n.	LC191235 (SSU), LC191250 (LSU)	Yamaguchi et al. (2016)
Apocalathium malmogiense (G.Sjöstedt) Craveiro, Daugbjerg, Moestrup & Calado (reference material)	SHTV1	Baltic Sea, off Finland: Uusimaa, Raseborg, Tvärminne (59°50'N, 23°15'E)	2002	A. Kremp s.n.	KF751923 (SSU+ITS+LSU)	Gottschling et al. (2005), Kremp et al. (2005), Logares et al. (2007a, 2008), Zinßmeister et al. (2012), Gottschling and Söhner (2013)
Archaeperidinium minutum (Kof.) Jørg.	n.inf.	eastern North Pacific, off Canada: British Columbia, Victoria	Mar 21, 2006	n.inf.	AB564309 (SSU), AB564310 (LSU)	Yamaguchi et al. (2011)
Blastodinium contortum Chatton [isolated from *Paracalanus* cf. *parvus* (Claus, 1863)]	n.inf.	eastern North Pacific, off USA–CA: Gulf of California, station 3 (24°14'N, 110°20'W)	Jun 11, 2008	n.inf.	FJ228701 (SSU+ITS+LSU)	Coats et al. (2008)

Blastodinium crassum Chatton [isolated from *Paracalanus* cf. *parvus* (Claus, 1863)]	n.inf.	eastern North Pacific, off USA–CA: Gulf of California, station 2 (24°13'N, 110°20'W)	Jun 10, 2008	n.inf.	FJ228702 (SSU+ITS+LSU)	Coats et al. (2008)
Caladoa arcachonensis Z.Luo, K.N.Mert. & H.Gu (holotype)	TIO278	eastern North Atlantic, Bay of Biscay, off France, Arcachon Bay (44°38'N, 1°04'W)	Apr, 2016	n.inf.	MK012071 (SSU), MK012081 (ITS), MK012076 (LSU)	Luo et al. (2019)
†*Calciodinellum operosum* Deflandre	SZN74	Mediterranean Sea, Tyrrhenian Sea, off Italy: Campania, Naples (40°43'N, 14°10'W)	n.inf.	M. Montresor	KF751922 (SSU+ITS+LSU)	D´Onofrio et al. (1999), Montresor et al. (2003), Gottschling and Plötner (2004), Kremp et al. (2005), Zinßmeister et al. (2012), Gottschling and Söhner (2013)
Duboscquodinium collinii Grassé [isolated from *Eutintinnus fraknoii* (Daday, 1887)]	VSM11	western Mediterranean Sea, off France: Alpes-Maritimes, Nice, Villefranche-sur-Mer (43°41'N, 7°19'E)	Sep 10, 2009	n.inf.	HM483399 (SSU+ITS+LSU)	Coats et al. (2010)
Durinskia oculata (F.Stein) Gert Hansen & Flaim (epitype)	GeoM*662 (≡ CCAC6039B, CCCM6005)	Czech Republic: Prague, Hlavní město Praha, Vltava (50°08'N, 14°23'E)	Sep, 2015	J. Kretschmann & M. Gottschling [J. Kretschmann] D043	KY693722 (SSU+ITS+LSU), KY693725 (LSUd8d10)	Kretschmann et al. (2018b), Žerdoner Čalasan et al. (2018)
Ensiculifera tyrrhenica (Balech) Zhun Li, K.N.Mert., H.Gu, Gottschling & H.H.Shin	GeoB*230	Mediterranean Sea, Ionian Sea, off Italy: Gulf of Taranto (40°07'N, 17°19'E)	Oct 26, 2002	D. Saracino [M. Kirsch] s.n.	HQ845329 (SSU+ITS+LSU)	Gottschling et al. (2005, 2008, 2012)
Gloeodinium montanum Klebs	CCAC0066	Germany: Hessen, Marburg, Nordeck	n.inf.	n.inf.	EF058238 (SSU), EF058258 (LSU)	Logares et al. (2007a), Moestrup and Daugbjerg (2007)

Herdmania litoralis J.D.Dodge		eastern North Pacific, off Canada, Boundary Bay: British Columbia, Centennial Beach	Mar 19, 2009	n.inf.	AB564300 (SSU), AB564306 (LSU)	Yamaguchi et al. (2011)
Heterocapsa arctica T.Horig. (holotype)	NCMA445 (≡ NCMA35)	North Atlantic, Baffin Bay (76°15'N, 82°33'W)	Jun 3, 1986 [Jul 28, 1989]	R. Selvin s.n.	KF925338 (SSU), JQ972677 (ITS), AY571372 (LSU)	Yoshida et al. (2003), Hansen and Daugbjerg (2004), Stern et al. (2012), Keeling et al. (2014), Preston & Gilg (unpubl. 2014)
Heterocapsa pseudotriquetra Iwataki, Gert Hansen & Fukuyo	GeoB 222	eastern North Atlantic, off Canary Islands (24°25'N, 17°11'W)	2003	[Meteor 58] [M. Kirsch]	AY499509 (ITS), MF423367 (LSU), MF423369 (LSUd8d10)	Gottschling et al. (2005), Tillmann et al. (2017)
Heterocapsa steinii Tillmann, Gottschling, Hoppenrath, Kusber & Elbr. (epitype)	UTKG7 (ITS clones 7, 9→14, LSU clones 14→15)	Baltic Sea, off Germany: Schleswig-Holstein, Kiel (54°19'N, 10°09'E)	Aug 7, 2013	A. Tillmann [U. Tillmann] s.n.	MF423350 (SSU), MF423353 (ITS), MF423362 (LSU)	Tillmann et al. (2017)
Islandinium minutum (Harland & P.C.Reid) Head	IMINCS2	Arctic Ocean (75°22'N, 176°19'E)	Aug 31, 2015	n.inf.	KY129807 (rRNA)	Potvin et al. (2018)
Johsia chumphonensis Z.Luo, Na Wang, K.N.Mert. & H.Gu	IshiMM4	Japan: Okinawa, Ishigaki Island, Nosoko (24.48N, 124.23E)	Jul 26, 2010	n.inf.	AB999980 (SSU), AB999984 (ITS), AB999988 (LSU)	Prabowo et al. (2017 unpubl.)
Kryptoperidinium cf. *triquetrum* (Ehrenb.) Tillmann, Gottschling, Elbr., Kusber & Hoppenrath	GeoB 459 (≡ CCAC4765B, CCCM327) (ribotype A)	Mediterranean Sea, Aegean Sea, off Greece: Peloponnese, Argolis, Nafplio, Nea Kios (37°35'N, 22°45'E)	Mar, 2010	C. Zinßmeister & S. Söhner [M. Kirsch] GRI00027	KY693721 (SSU+ITS+LSU), KY693724 (LSUd8d10)	Kretschmann et al. (2018b), Žerdoner Čalasan et al. (2018)

Matsuokea aff. *loeblichii* (El.R.Cox & H.J.Arn.) Zhun Li, K.N.Mert., H.Gu & H.H.Shin	GeoB*220	eastern South Atlantic, off Namibia (30°30'S, 13°22'E)	2003	[Meteor 57] [M. Kirsch]	HQ845328 (SSU+ITS+LSU)	Gottschling et al. (2005, 2008, 2012)
Naiadinium polonicum (Wołosz.) Carty (reference material)	HBI:MG200823a	n.inf.	n.inf.	n.inf.	JQ639764 (SSU), JQ639772 (ITS), JQ639754 (LSU)	Zhang et al. (unpubl. 2012)
Pachena abriliae A.Reñé, Satta & Hoppenrath	Castelldefels	westren Mediterranean Sea, off Spain: Barcelona, Castelldefels (41°16'N, 1°56'E)	2017	A. Reñé s.n.	MN707940 (SSU), MN703810 (LSU)	Hoppenrath et al. (2020)
Palatinus apiculatus (Ehrenb.) Craveiro, Calado, Daugbjerg & Moestrup (epitype)	GeoM*762 (≡ CCAC6788B)	Germany: Berlin, Mitte, Tiergarten (52°31'N, 13°21'E)	Mar 28, 2016	M. Gottschling [J. Kretschmann] D047	KY996787 (SSU+ITS+LSU), MG255412 (LSUd8d10)	Kretschmann et al. (2018c)
Parvodinium trawinskii Kretschmann, Owsianny, Zerdoner & Gottschling (holotype)	GeoM*753 (≡ CCAC6787B)	Poland: Lesser Poland, Tatra, Długi Staw Gąsienicowy (49°14'N, 20°01'E, 1784m)	Sep 22, 2015	P.M. Owsianny, K. Trawiński & G. Marciniak [J. Kretschmann] PL019	MG255427 (SSU+ITS+LSU), MG255419 (LSUd8d10)	Kretschmann et al. (2018a)
Parvodinium cf. *umbonatum* (F.Stein) Carty	GeoM*795 (≡ CCAC6789B)	Poland: Lesser Poland, Tatra, Toporowy Staw Niżni (49°17'N, 20°02'E)	Aug 4, 2016	J. Kretschmann & P.M. Owsianny [J. Kretschmann] PL069	MG255428 (SSU+ITS+LSU), MG255420 (LSUd8d10)	Kretschmann et al. (2018a)
Pentapharsodinium cf. *imariense* (S.Kobayashi & Matsuoka) Zhun Li, K.N. Mert., H. Gu, Gottschling & H.H. Shin	GeoB 284 (≡ CCAC4747B, CCAP1111/1)	North Sea, off Norway: Sør-Trøndelag, Snillfjord, Åstfjorden, Mjønos (harbour) (63°28'N, 9°25'E)	Oct 27, 2003	M. Gottschling & M. Petersen [M. Kirsch] SCA00010	KR362906 (SSU), AY728076 (ITS), DQ167856 (LSU)	Gottschling et al. (2005, 2008), Gottschling and Söhner (2013)

Peridiniopsis borgei Lemmerm. (reference material)	PBSK-A	Sweden: Skåne, St. Kalkbrottsdammen (55°31'N, 12°55'E)	2005	n.inf.	EF058241 (SSU), EU445295 (ITS), EF058261 (LSU), FJ236464 (LSU)	Logares et al. (2007b, 2009), Craveiro et al. (2009)
"*Peridinium*" *americanum* Gran & Braarud	080218-12	western North Pacific, East China Sea, off Japan: Kyūshū, Nagasaki, Sasebo	n.inf.	n.inf.	AB716911 (SSU), AB716925 (LSU)	Matsuoka and Kawami (2013)
Peridinium bipes forma *globosum* Er.Lindem.	NIES495 (≡ LOND9)	Japan: Fukushima, Lake Onogawa	Jul 30, 1985	[T. Sawaguchi s.n.]	GU046392 (SSU+ITS+LSU)	Ki et al. (2011), Ki and Han (2007c)
Peridinium bipes forma *occultatum* (Er.Lindem.) M.Lefèvre	HYJA0310	eastern Indian Ocean, off South Korea: Juam	n.inf.	n.inf.	GU046390 (SSU+ITS+LSU)	Ki et al. (2011), Ki and Han (2007c)
Peridinium cinctum (O.F.Müll.) Ehrenb.	CCAC0102 (≡ M1576/1)	Germany: Lower Saxony, Wittmund, Spiekeroog	1998	[D. Hille s.n.]	EF058244 (SSU), KF751925 (SSU+ITS+LSU), MF423370 (LSUd8d10)	Gottschling et al. (2005, 2017), Logares et al. (2007b), Zinßmeister et al. (2012), Gottschling and Söhner (2013)
Peridinium willei Huitf.-Kaas	TK007	Japan: Hokkaidō, Kiritappu-shitsugen	n.inf.	n.inf.	AB232669 (SSU+ITS+LSU)	Takano and Horiguchi (2006)
†*Pernambugia tuberosa* (Kamptner) Janofske & Karwath	GeoB*61 (≡ CCAC4752B, CCAP1141/1)	western South Atlantic (11°32'S, 28°35'W, −100m)	Feb 27, 1997	[Meteor 38/1] [M. Kirsch] 4321-9	KR362907 (SSU), JN982372 (ITS+LSU), MF423371 (LSUd8d10)	Gottschling et al. (2005, 2008, 2017), Zinßmeister et al. (2012), Gottschling and Söhner (2013)
Pfiesteria piscicida Steid. & J.M.Burkh.	n.inf.	USA–MD: Chicamacomico River	1997	K.A. Steidinger & J.M. Burkholder s.n.	AY112746 (rRNA)	Saito et al. (2002)
†*Posoniella tricarinelloides* (G.Versteegh) Streng, Banasová, Reháková & H.Willems	PTLY01	western North Pacific, Yellow Sea, off China: Jiangsu, Lianyungang (34°49'N, 119°32E, −15m)	May 9, 2011	H. Gu s.n.	KC511790 (SSU), KC511792 (ITS), KC511794 (LSU)	Gu et al. (2013)

Preperidinium meunieri (Pavill.) Elbr.	cell1_3clones	western North Atlantic, off USA–MA: Salt Pond	n.inf.	n.inf.	EF152930 (LSU)	Gribble and Anderson (2007)
Protoperidinium bipes (Paulsen) Balech	n.inf.	western North Pacific, Sea of Japan, off Japan: Hokkaidō, Ishikari	n.inf.	n.inf.	AB284159 (SSU), AB284160 (LSU)	Yamaguchi et al. (2007)
Protoperidinium pellucidum Bergh	#28	western North Pacific, Sea of Japan, off Japan: Hokkaidō, Shiribeshi, Otaru (43°10'N, 141°01'E)	Oct 31, 2002	n.inf.	AB181903 (SSU), AB255862 (LSU)	Yamaguchi and Horiguchi (2005), Yamaguchi et al. (2006)
Scrippsiella bicarinata Zinssmeister, S.Soehner, S.Meier & Gottschling	GeoB 411 (≡ CCAC5106B, CCCM324)	Mediterranean Sea, Tyrrhenian Sea, off Italy: Formia (41°15'N, 13°36'E)	Apr, 2009	M. Gottschling, C. Zinßmeister, S. Söhner [M. Kirsch] ITA00044	MH497031 (SSU), KF751927 (SSU+ITS+LSU)	Zinßmeister et al. (2012), Gottschling and Söhner (2013), Kretschmann et al. (2018a)
"*Scrippsiella*" *donghaienis* H.Gu	CS-168	eastern Indian Ocean, off Australia (33°S, 138°E)	1983	J.L. Stauber s.n.	JN982374 (ITS+LSU), MH497032 (LSUd8d10)	Gottschling et al. (2005), Stern et al. (2012), Zinßmeister et al. (2012), Žerdoner Čalasan et al. (2019)
Scrippsiella sweeneyae Balech	CCCM280	n.inf.	n.inf.	A. Chan s.n.	HQ845331 (SSU+ITS+LSU)	Gottschling et al. (2005, 2012), Kremp et al. (2005)
Theleodinium calcisporum Craveiro, Pandeirada, Daugbjerg, Moestrup & Calado (holotype)	MP69	Portugal: Aveiro, Ílhavo, Gafanha da Boavista (40°36'N, 8°42'W)	Mar 2, 2011	n.inf.	KC699492 (SSU+ITS+LSU)	Craveiro et al. (2013)
Thoracosphaera heimii (Lohmann) Kamptner	CCCM670	western North Atlantic, Gulf of Mexico	Apr 23, 1980	L. Brand s.n.	HQ845327 (SSU+ITS+LSU)	Saldarriaga Echavarría et al. (2001), Gottschling et al. (2012), Zinßmeister et al. (2012)

Tintinnophagus acutus Coats (isolated from *Tintinnopsis cylindrica* Daday, 1887) (holotype)	n.inf.	western North Atlantic, off USA–MD: Rhode River, Chesapeake Bay (38°53'N, 76°33'W)	n.inf.	n.inf.	HM483397 (SSU+ITS+LSU)	Coats et al. (2010)
Unruhdinium penardii (Lemmerm.) Gottschling (reference material)	Jiulongjiang	China: Fujian, Zhangzhou, Jiulongjiang River (24°35'N, 117°41'E)	Feb, 2009	n.inf.	HM596543 (SSU), HM596551 (ITS), HM596556 (LSU)	Zhang et al. (2011)
Vulcanodinium rugosum Nézan & Chomérat	G	France	n.inf.	n.inf.	MG826115 (SSU), MG826367 (ITS), MG826107 (LSU)	Luo et al. (2018)
Zooxanthella nutricula K.Brandt (isolated from *Thalassicolla nucleata* Huxley, 1851)	BBSR323	western North Atlantic, Sargasso Sea, off UK, the Bermudas: 3-5 miles SE of Bermuda	n.nf.	n.nf.	U52356 (SSU), KC511788 (ITS+LSU)	Gast and Caron (1996), Gottschling and McLean (2013)
<u>Gonyaulacales</u>						
Alexandrium affine (H.Inoue & Fukuyo) Balech	AC-1	western North Pacific, South China Sea	n.inf.	n.inf.	JF906996 (rRNA)	Tang et al. (2012)
Alexandrium lusitanicum Balech	NCMA113 (≡ AL, AL1V)	eastern North Atlantic, off Spain: Ria de Vigo (42°14'N, 8°48'W)	Sep 1, 1987	I. Bravo [I. Bravo] s.n.	AY831408 (rRNA)	Ki and Han (2005), Rogers et al. (2006), McCauley et al. (2009)
Alexandrium pacificum Litaker	Axsp-K05	eastern Indian Ocean, off southern South Korea	n.inf.	n.inf.	DQ785887 (rRNA)	Ki and Han (2007b)
Ceratium furcoides (Levander) Langhans	HBI:SC201002a	n.inf.	n.inf.	n.inf.	JQ639757 (SSU), JQ639769 (ITS), JQ639748 (LSU)	Zhang et al. (unpubl. 2012)
Ceratocorys horrida F.Stein	GeoB 183	western South Atlantic (26°22'S, 38°53'W, –5m)	Mar 9, 2000	K.J.S. Meier & M. Streng [Meteor 46/4] [M. Kirsch] WP 3/9/b	MK405481 (SSU+ITS+LSU), MK405482 (LSUd8d10)	Gottschling et al. (2020)

Gonyaulax elongata (P.C.Reid) Ellegaard, Daugbjerg, Rochon, Jane Lewis & I.Harding	cyst 6	Arctic Ocean, off Canada (70°17'N, 135°28'W)	Sep 1, 2014	n.inf.	KU358947 (SSU+ITS+LSU)	Van Nieuwenhove (unpubl. 2015)
Gonyaulax cf. *membranacea* (M.Rossignol) Ellegaard, Daugbjerg, Rochon, Jane Lewis & I.Harding	NCMA409 (≡ W1)	western North Atlantic, off USA–ME: West Boothbay Harbor, Bigelow (43°51'N, 69°38'W)	Oct 10, 1986	R. Lande [R. Lande] s.n.	AF022155 (SSU), EU532487 (ITS), EU532478 (LSU)	Saunders et al. (1997), Howard et al. (2009)
Impagidinium caspienense Marret	KC18	Caspian Sea, off Iran: Gilan (37°31'N, 49°55'E)	Dec 3, 2011	S. Bagheri s.n.	LC222300 (SSU), LC222301 (ITS), LC222302 (LSU)	Mertens et al. (2017)
Lingulodinium cf. *polyedra* (F.Stein) J.D.Dodge	LPCQ1	eastern North Pacific, Gulf of California, Bahía Concepción, off Mexico	2000	[L. Morquecho] s.n.	JQ616824 (SSU+ITS+LSU), JQ616830 (LSU)	Herrera Sepúlveda et al. (2013)
Protoceratium reticulatum (Clap. & J.Lachm.) Buetschli	n.inf.	North Sea, Kattegat, off Sweden (57°30'N, 11°48'E)	May, 2010	n.inf.	AB727655 (SSU+ITS+LSU)	Mertens et al. (2012)
Pyrodinium bahamense L.Plate	Mas9603-Pbc	western North Pacific, South China Sea, off Philippines, Masinloc Bay	Mar, 1996	n.inf.	AB936751 (SSU), AB936755 (LSU)	Iwataki & Matsuoka (unpubl. 2014)
Pyrrhotriadinium polyedricum (C.H.G.Pouchet) Nakada	G1	western South Atlantic, off Brazil: São Paulo, Ubatuba (23°33'S, 45°07'W)	Feb 28, 2014	F. Gómez s.n.	KM886380 (SSU+ITS+LSU)	Gómez et al. (2015b)
†*Pyxidinopsis psilata* (D.Wall & B.Dale) M.J.Head	LH3	Caspian Sea, off Iran (37°31N, 49°55'E, –25m)	Sep 3, 2011	S. Bagheri s.n.	KY681700 (SSU), MT041629 (ITS), MT039439 (LSU)	Mertens et al. (2017), Zhang et al. (2020)
Sourniaea diacantha (Meunier) H.Gu, K.N.Mert., Zhun Li & H.H.Shin	TIO492	eastern North Pacific, off Canada, Boundary Bay: British Columbia (48°26'N, 123°28'W)	Apr 20, 2017	n.inf.	MT039422 (SSU), MT041622 (ITS), MT039429 (LSU)	Zhang et al. (2020)

Spiniferites belerius P.C.Reid	KC58	Japan: Hokkaidō, Abashiri, Lake Saroma (44°07'N, 143°52'E, –18m)	Jul 22, 2011	Y. Takano & K.N. Mertens s.n.	LC222309 (SSU), LC222310 (LSU)	Mertens et al. (2017)
†*Spiniferites ramosus* (Ehrenb.) Mantell	KC51	eastern North Pacific, Strait of Georgia, off Canada (49°02'N, 123°26'W, –300m)	Oct 2, 2011	V. Pospelova s.n.	LC222307 (SSU), LC222308 (LSU)	Mertens et al. (2017)
Thecadinium kofoidii (Herdman) J.Larsen	SCCAP K-1504	North Sea, off Germany: Helgoland (54°12'N, 7°54'E)	Aug, 2002	M.Hoppenrath [M. Hoppenrath] s.n,	GU295204 (SSU), JX262497 (ITS), GU295207 (LSU)	Hoppenrath and Leander (2010), Orr et al. (2012)
Thecadinium cf. *kofoidii* (Herdman) J.Larsen	Th.cf.k-2	western North Pacific, Sea of Japan, Vostok Bay, Reef Bay, off Russia (42°51'N, 132°37'E)	Aug 9, 2016	n.inf.	KY575447 (SSU), KY575448 (ITS), KY575445 (LSU), KY575446 (LSUd8d10)	Selina et al. (2019)
"*Thecadinium*" *yashimaense* Yoshimatsu, Toriumi & J.D.Dodge	NCMA1890 (≡ CCCM682)	eastern North Pacific, Boundary Bay, off Canada, Boundary Bay: British Columbia (49°00'N, 123°00'W)	Apr, 1988	D. Jacobsen [E. Simons] s.n.	AY238477 (SSU), FJ823640 (ITS), GU295209 (LSU)	Hoppenrath et al. (2004), Lin et al. (2006a), Hoppenrath and Leander (2010), Leblond et al. (2010), Stern et al. (2012)
Tripos furca (Ehrenb.) F.Gómez	n.inf.	western North Pacific, Yellow Sea, off China: Liaoling, Huludao	n.inf.	n.inf.	AJ276699 (SSU), AJ276700 (ITS)	Zhuang et al. (2001)
Tripos longipes (Bailey) F.Gómez	NCMA1770	western North Atlantic, off USA–ME: West Boothbay Harbor, Bigelow (43°51'N, 69°38'W)	Jan 9, 1997	S.L. Morton [S.L. Morton] s.n.	DQ388462 (SSU), EU927566 (ITS), EU165305 (LSU)	Lin et al. (2006a), Scorzetti et al. (2009) Ferrell & Beaton (unpubl. 2008)

<u>Dinophysales</u>

Amphisolenia bidentata Schröd. (chimeric as used in Orr et al. 2012)	LE392	Indian Ocean, off Australia (16°02'S, 119°20'E)	fall 2006	n.inf.	GU196149 (SSU), FJ808682 (LSU)	Jensen and Daugbjerg (2009), Daugbjerg et al. (2013)
Dinophysis caudata Kent	FTL69	western North Atlantic, off USA–FL: Ft. Lauderdale (26°05'N, 80°03'W)	Feb 26, 2008	n.inf.	EU780644 (SSU+ITS+LSU)	Handy et al. (2009)
Histioneis sp.	FTL62	western North Atlantic, off USA–FL: Ft. Lauderdale (26°05'N, 80°03'W)	Feb 26, 2008	n.inf.	EU780646 (SSU+ITS+LSU)	Handy et al. (2009)
Ornithocercus magnificus F.Stein	CBC4L7	western North Atlantic, off USA–VA: shelf break off lower Chesapeake Bay (36°20'N, 74°44'W)	Oct 15, 2007	n.inf.	EU780649 (SSU+ITS+LSU)	Handy et al. (2009)
Phalacroma rapa Jørg.	CBC4L5	western North Atlantic, off USA–VA: shelf break off lower Chesapeake Bay (36°20'N, 74°44'W)	Oct 15, 2007	n.inf.	EU780655 (SSU+ITS+LSU)	Handy et al. (2009)
Phalacroma cf. *rotundatum* (Clap. & J.Lachm.) Kof. & J.R.Michener	FTL121	western North Atlantic, off USA–FL: Ft. Lauderdale (26°05'N, 80°03'W)	Feb 26, 2008	n.inf.	EU780657 (SSU+ITS+LSU)	Handy et al. (2009)
<u>Prorocentrales</u>						
Adenoides eludens (Herdman) Balech	ADE2	eastern North Atlantic, off France: Hauts-de-France, Wimereux (50º46'N, 1º37'E)	Jun, 2011	n.inf.	LC002839 (SSU), LC002844 (ITS+LSU)	Gómez et al. (2015a)
Prorocentrum donghaiense D.D.Lu (chimeric)	n.inf.	western North Pacific, East China Sea	2002	S. Lin s.n.	DQ336054 (SSU), AY465116 (ITS), AY822610 (LSU), DQ336055 (cyb)	Zhang et al. (2004), Lin et al. (2006b)

Prorocentrum hoffmannianum M.A.Faust	NCMA683 (≡ PL200A)	western North Atlantic, off USA–FL: Knight Key (24°42'N, 81°08'W)	Nov 1, 1985	J. Bomber [J. Bomber] s.n.	KF885225 (SSU+ITS+LSU), KC622310 (SSU+ITS+LSU)	Lee (unpubl. 2013), Herrera Sepúlveda et al. (2015)
Prorocentrum leve M.A.Faust, Kibler, Vandersea, P.A.Tester & Litaker (holotype)	NCMA2634	western North Atlantic, Caribbean Sea, off Belize: Stann Creek, Twin Cays (16°50N, 88°06'W)	May 21, 2002	S. Kibler [S. Kibler] s.n.	DQ238043 (SSU+ITS+LSU)	Faust et al. (2008)
Prorocentrum lima (Ehrenb.) F.Stein	NCMA685 (≡ PL2V)	eastern North Atlantic, off Spain: Ria de Vigo (42°14'N, 8°48'W)	n.inf.	I. Bravo s.n.	AB189765 (ITS), DQ336179 (LSU)	Nagahama et al. (2011)
Prorocentrum lima (Ehrenb.) F.Stein	NCMA1370 (≡ FIT#69)	western North Atlantic, off USA–FL: Knight Key (24°42'N, 81°08'W)	n.inf.	J. Bomber s.n.	EU927507 (ITS), DQ336180 (LSU)	Ferrell & Beaton (unpubl. 2008), Murray et al. (2009)
Prorocentrum cf. *ima* (Ehrenb.) F.Stein	SKLMP_W074	western North Pacific, South China Sea			MK547120 (SSU), MK605056 (ITS), MK544035 (LSUd8d10)	Yiu et al. (unpubl. 2020)
Prorocentrum micans Ehrenb.	NCMA1589 (≡ 992M3)	USA–RI: Narragansett Bay (41°36'N, 71°24'W)	Sep 19, 1992	P. Hargraves [P. Hargraves] s.n.	EU780638 (SSU+ITS+LSU)	Zhang et al. (2008), Handy et al. (2009)
Prorocentrum minimum (Pavill.) J.Schiller	D-127	eastern Indian Ocean, off South Korea: Tongyeong	n.inf.	n.inf.	JX402086 (rRNA)	Cheon & Ki (unpubl. 2013)
Pseudadenoides kofoidii (Herdman) F.Gómez, R.Onuma, Artigas & T.Horig.	PSE6	eastern North Atlantic, off France: Hauts-de-France, Wimereux (50°46'N, 1°37'E)	Jun, 2011	n.inf.	LC002843 (SSU), LC002848 (ITS+LSU)	Gómez et al. (2015a)

References

Al-Kandari, M. A., A. C. Highfield, M. J. Hall, P. Hayes, and D. C. Schroeder. 2011. Molecular tools separate harmful algal bloom species, *Karenia mikimotoi*, from different geographical regions into distinct sub-groups. Harmful Algae 10:636–643.

Annenkova, N. V. 2018. Identification of Lake Baikal plankton dinoflagellates from the genera *Gyrodinium* and *Gymnodinium* using single-cell PCR. Russian Journal of Genetics 54:1302–1313.

Bachvaroff, T. R., S. Kim, L. Guillou, C. F. Delwiche, and D. W. Coats. 2012. Molecular diversity of the syndinean genus *Euduboscquella* based on single-cell PCR analysis. Applied and Environmental Microbiology 78:334–345.

Boutrup, P. V., Ø. Moestrup, U. Tillmann, and N. Daugbjerg. 2017. Ultrastructure and phylogeny of *Kirithra asteri* gen. et sp. nov. (Ceratoperidiniaceae, Dinophyceae) — A free-living, thin-walled marine photosynthetic dinoflagellate from Argentina. Protist 168:586–611.

Brummitt, R. K., and C. E. Powell. 1992. Authors of plant names: A list of authors of scientific names of plants, with recommended standard forms of their names, including abbreviations. Royal Botanic Gardens, Kew.

Carlos, A. A., B. K. Baillie, M. Kawachi, and T. Maruyama. 1999. Phylogenetic position of *Symbiodinium* (Dinophyceae) isolates from tridacnids (Bivalvia), cardiids (Bivalvia), a sponge (Porifera), a soft coral (Anthozoa), and a free-living strain. Journal of Phycology 35:1054–1062.

Coats, D. W., T. R. Bachvaroff, S. M. Handy, S. Kim, I. Gárate-Lizárraga, and C. F. Delwiche. 2008. Prevalence and phylogeny of parasitic dinoflagellates (genus *Blastodinium*) infecting copepods in the Gulf of California. CICIMAR Oceánides 23:67–77.

Coats, D. W., S. Kim, T. R. Bachvaroff, S. M. Handy, and C. F. Delwiche. 2010. *Tintinnophagus acutus* n. g., n. sp. (Phylum Dinoflagellata), an ectoparasite of the ciliate *Tintinnopsis cylindrica* Daday 1887, and its relationship to *Duboscquodinium collini* Grassé 1952. Journal of Eukaryotic Microbiology 57:468–482.

Craveiro, S. C., A. J. Calado, N. Daugbjerg, and Ø. Moestrup. 2009. Ultrastructure and LSU rDNA-based revision of *Peridinium* group Palatinum (Dinophyceae) with the description of *Palatinus* gen. nov. Journal of Phycology 45:1175–1194.

Craveiro, S. C., M. S. Pandeirada, N. Daugbjerg, Ø. Moestrup, and A. J. Calado. 2013. Ultrastructure and phylogeny of *Theleodinium calcisporum* gen. et sp. nov., a freshwater dinoflagellate that produces calcareous cysts. Phycologia 52:488–507.

D´Onofrio, G., D. Marino, L. Bianco, E. Busico, and M. Montresor. 1999. Toward an assessment on the taxonomy of dinoflagellates that produce calcareous cysts (Calciodinelloideae, Dinophyceae): A morphological and molecular approach. Journal of Phycology 35:1063–1078.

Daugbjerg, N., G. Hansen, J. Larsen, and Ø. Moestrup. 2000. Phylogeny of some of the major genera of dinoflagellates based on ultrastructure and partial LSU rDNA sequence data, including the erection of three new genera of unarmoured dinoflagellates. Phycologia 39:302–317.

Daugbjerg, N., M. H. Jensen, and P. J. Hansen. 2013. Using nuclear-encoded LSU and SSU rDNA sequences to identify the eukaryotic endosymbiont in *Amphisolenia bidentata* (Dinophyceae). Protist 164:411–422.

Faust, M. A., M. W. Vandersea, S. R. Kibler, P. A. Tester, and R. W. Litaker. 2008. *Prorocentrum levis*, a new benthic species (dinophyceae) from a mangrove island, Twin Cays, Belize. Journal of Phycology 44:232–240.

Fawcett, R. C., and M. W. Parrow. 2012. Cytological and phylogenetic diversity in freshwater *Esoptrodinium*/*Bernardinium* species (Dinophyceae). Journal of Phycology 48:793–807.

Garces, E., M. Fernandez, A. Penna, K. Van Lenning, A. Gutierrez, J. Camp, and M. Zapata. 2006. Characterization of NW Mediterranean *Karlodinium* spp. (Dinophyceae) strains using morphological, molecular, chemical, and physiological methodologies. Journal of Phycology 42:1096–1112.

Gast, R. J., and D. A. Caron. 1996. Molecular phylogeny of symbiotic dinoflagellates from planktonic Foraminifera and Radiolaria. Molecular Biology and Evolution 13:1192–1197.

Gómez, F., R. Onuma, L. F. Artigas, and T. Horiguchi. 2015a. A new definition of *Adenoides eludens*, an unusual marine sand-dwelling dinoflagellate without cingulum, and *Pseudadenoides kofoidii* gen. & comb. nov for the species formerly known as *Adenoides eludens*. European Journal of Phycology 50:125–138.

Gómez, F., D. Qiu, R. M. Lopes, and S. Lin. 2015b. *Fukuyoa paulensis* gen. et sp. nov., a new genus for the globular species of the dinoflagellate *Gambierdiscus* (Dinophyceae). PLoS One 10:e0119676.

Gómez, F., and A. Skovgaard. 2014. Molecular phylogeny of the parasitic dinoflagellate *Chytriodinium* within the *Gymnodinium* clade (Gymnodiniales, Dinophyceae). Journal of Eukaryotic Microbiology 62:422–425.

Gottschling, M., J. Chacón, A. Žerdoner Čalasan, St. Neuhaus, J. Kretschmann, H. Stibor, and U. John. 2020. Phylogenetic placement of environmental sequences using taxonomically reliable databases helps to rigorously assess dinophyte biodiversity in Bavarian lakes (Germany). Freshwater Biology 65:193–208.

Gottschling, M., H. Keupp, J. Plötner, R. Knop, H. Willems, and M. Kirsch. 2005. Phylogeny of calcareous dinoflagellates as inferred from ITS and ribosomal sequence data. Molecular Phylogenetics and Evolution 36:444–455.

Gottschling, M., J. Kretschmann, and A. Žerdoner Čalasan. 2017. Description of Peridiniopsidaceae, fam. nov. (Peridiniales, Dinophyceae). P hytotaxa 299:293–296.

Gottschling, M., and T. I. McLean. 2013. New home for tiny symbionts: Dinophytes determined as *Zooxanthella* are Peridiniales and distantly related to *Symbiodinium*. Molecular Phylogenetics and Evolution 67:217–222.

Gottschling, M., and J. Plötner. 2004. Secondary structure models of the nuclear Internal Transcribed Spacer regions and 5.8S rRNA in Calciodinelloideae (Peridiniaceae) and other dinoflagellates. Nucleic Acids Research 32:307–315.

Gottschling, M., S. S. Renner, K. J. S. Meier, H. Willems, and H. Keupp. 2008. Timing deep divergence events in calcareous dinoflagellates. Journal of Phycology 44:429–438.

Gottschling, M., and S. Söhner. 2013. An updated list of generic names in the Thoracosphaeraceae. Microorganisms 1:122–136.

Gottschling, M., S. Söhner, C. Zinßmeister, U. John, J. Plötner, M. Schweikert, K. Aligizaki, and M. Elbrächter. 2012. Delimitation of the Thoracosphaeraceae (Dinophyceae), including the calcareous dinoflagellates, based on large amounts of ribosomal RNA sequence data. Protist 163:15–24.

Gribble, K. E., and D. M. Anderson. 2007. High intraindividual, intraspecific, and interspecific variability in large-subunit ribosomal DNA in the heterotrophic dinoflagellates *Protoperidinium*, *Diplopsalis*, and *Preperidinium* (Dinophyceae). Phycologia 46:315–324.

Gu, H., M. Kirsch, C. Zinßmeister, S. Söhner, K. J. S. Meier, T. Liu, and M. Gottschling. 2013. Waking the dead: Morphological and molecular characterization of extant †*Posoniella tricarinelloides* (Thoracosphaeraceae, Dinophyceae). Protist 164:583–597.

Gu, H., Z. Luo, K. N. Mertens, A. M. Price, R. E. Turner, and N. N. Rabalais. 2015. Cyst-motile stage relationship, morphology, ultrastructure, and molecular phylogeny of the gymnodinioid dinoflagellate *Barrufeta resplendens* comb. nov., formerly known as *Gyrodinium resplendens*, isolated from the Gulf of Mexico. Journal of Phycology 51:990–999.

Handy, S. M., T. R. Bachvaroff, R. E. Timme, D. W. Coats, S. Kim, and C. F. Delwiche. 2009. Phylogeny of four Dinophysiacean genera (Dinophyceae, Dinophysiales) based on rDNA sequences from single cells and environmental samples. Journal of Phycology 45:1163–1174.

Hansen, G., and N. Daugbjerg. 2004. Ultrastructure of *Gyrodinium spirale*, the type species of *Gyrodinium* (Dinophyceae), including a phylogeny of *G. dominans*, *G. rubrum* and *G. spirale* deduced from partial LSU rDNA sequences. Protist 155:271–294.

Hansen, G., N. Daugbjerg, and P. Henriksen. 2000. Comparative study of *Gymnodinium mikimotoi* and *Gymnodinium aureolum*, comb. nov. (= *Gyrodinium aureolum*) based on morphology, pigment composition, and molecular data. Journal of Phycology 36:394–410.

Hansen, G., N. Daugbjerg, and P. Henriksen. 2007. *Baldinia anauniensis* gen. et sp. nov.: A 'new' dinoflagellate from Lake Tovel, N. Italy. Phycologia 46:86–108.

Hansen, G., N. Daugbjerg, and Ø. Moestrup. 2018. The rainwater rock-pool dinoflagellate *Nottbeckia ochracea* gen. et comb. nov (syn.: *Hemidinium ochraceum*) - A fine-structural and molecular study with emphasis on the motile stage. Protist 169:280–306.

Henrichs, D. W., H. M. Sosik, R. J. Olson, and L. Campbell. 2011. Phylogenetic analysis of *Brachidinium capitatum* (Dinophyceae) from the Gulf of Mexico indicates membership in the Kareniaceae. Journal of Phycology 47:366–374.

Herrera Sepúlveda, A., N. Y. Hernandez-Saavedra, L. K. Medlin, and N. West. 2013. Capillary electrophoresis finger print technique (CE-SSCP): An alternative tool for the monitoring activities of HAB species in Baja California Sur Costal. Environmental Science and Pollution Research 20:6863–6871.

Herrera Sepúlveda, A., L. K. Medlin, G. Murugan, A. P. Sierra-Beltrán, A. A. Cruz-Villacorta, and N. Y. Hernández-Saavedra. 2015. Are *Prorocentrum hoffmannianum* and *Prorocentrum belizeanum* (Dinophyceae, Prorocentrales), the same species? An integration of morphological and molecular data. Journal of Phycology 51:173–188.

Hoppenrath, M., T. R. Bachvaroff, S. M. Handy, C. F. Delwiche, and B. S. Leander. 2009. Molecular phylogeny of ocelloid-bearing dinoflagellates (Warnowiaceae) as inferred from SSU and LSU rDNA sequences. BMC Evolutionary Biology 9:116.

Hoppenrath, M., and B. S. Leander. 2010. Dinoflagellate phylogeny as inferred from Heat Shock Protein 90 and ribosomal gene sequences. PLoS One 5:e13220.

Hoppenrath, M., S. A. Murray, S. F. Sparmann, and B. S. Leander. 2012. Morphology and molecular phylogeny of *Ankistrodinium* gen. nov. (Dinophyceae), a new genus of marine sand-dwelling dinoflagellates formerly classified within *Amphidinium*. Journal of Phycology 48:1143–1152.

Hoppenrath, M., A. Reñé i Vicente, C. T. Satta, A. Yamaguchi, and B. S. Leander. 2020. Morphology and molecular phylogeny of a new marine, sand-dwelling dinoflagellate genus, *Pachena* (Dinophyceae), with descriptions of three new species. Journal of Phycology 56:798–817.

Hoppenrath, M., J. F. Saldarriaga Echavarría, M. Schweikert, M. Elbrächter, and F. J. R. Taylor. 2004. Description of *Thecadinium mucosum* sp. nov. (Dinophyceae), a new sand-dwelling marine dinoflagellate, and an emended description of *Thecadinium inclinatum* Balech. Journal of Phycology 40:946–961.

Howard, M. D. A., G. J. Smith, and R. M. Kudela. 2009. Phylogenetic relationships of yessotoxin-producing dinoflagellates, based on the large subunit and Internal Transcribed Spacer ribosomal DNA domains. Applied and Environmental Microbiology 75:54–63.

Hu, Z., Y. Deng, Z. Luo, L. Shang, F. Kong, H. Gu, Z. Zhao, and Y. Z. Tang. 2020. Characterization of the unarmored dinoflagellate *Pseliodinium pirum* (Ceratoperidiniaceae) from Jiaozhou Bay, China. Phycological Research 68:3–13.

Ishitani, Y., Y. Ujiie, and K. Takishita. 2014. Uncovering sibling species in Radiolaria: Evidence for ecological partitioning in a marine planktonic protist. Molecular Phylogenetics and Evolution 78:215–222.

Janouškovec, J., A. Horák, M. Oborník, J. Lukeš, and P. J. Keeling. 2010. A common red algal origin of the apicomplexan, dinoflagellate, and heterokont plastids. Proceedings of the National Academy of Sciences of the United States of America 107:10949–10954.

Jensen, M. H., and N. Daugbjerg. 2009. Molecular phylogeny of selected species of the order Dinophysiales (Dinophyceae)—Testing the hypothesis of a dinophysioid radiation. Journal of Phycology 45:1136–1152.

Jeong, H. J., S. H. Jang, Ø. Moestrup, N. S. Kang, S. Y. Lee, É. Potvin, and J. H. Noh. 2014a. *Ansanella granifera* gen. et sp. nov (Dinophyceae), a new dinoflagellate from the coastal waters of Korea. Algae 29:75–99.

Jeong, H. J., S. Y. Lee, N. S. Kang, Y. D. Yoo, A. S. Lim, M. J. Lee, H. S. Kim, W. Yih, H. Yamashita, and T. C. LaJeunesse. 2014b. Genetics and morphology characterize the dinoflagellate *Symbiodinium voratum*, n. sp., (Dinophyceae) as the sole representative of *Symbiodinium* clade E. Journal of Eukaryotic Microbiology 61:75–94.

Jørgensen, M. F., S. A. Murray, and N. Daugbjerg. 2004. A new genus of athecate interstitial dinoflagellates, *Togula* gen. nov., previously encompassed within *Amphidinium sensu lato*: Inferred from light and electron microscopy and phylogenetic analyses of partial large subunit ribosomal DNA sequences. Phycological Research 52:284–299.

Kang, N. S., H. J. Jeong, Ø. Moestrup, T. Y. Jang, S. Y. Lee, and M. J. Lee. 2015. *Aduncodinium* gen. nov and *A. glandula* comb. nov (Dinophyceae, Pfiesteriaceae), from coastal waters off Korea: Morphology and molecular characterization. Harmful Algae 41:25–37.

Kang, N. S., H. J. Jeong, Ø. Moestrup, and T. G. Park. 2011. *Gyrodiniellum shiwhaense* n. gen., n. sp., a new planktonic heterotrophic dinoflagellate from the coastal waters of Western Korea: Morphology and ribosomal DNA gene sequence. Journal of Eukaryotic Microbiology 58:284–309.

Keeling, P. J., F. Burki, H. M. Wilcox, B. Allam, E. E. Allen, L. A. Amaral-Zettler, E. V. Armbrust, J. M. Archibald, A. K. Bharti, C. J. Bell, B. Beszteri, K. D. Bidle, C. T. Cameron, L. Campbell, D. A. Caron, R. A. Cattolico, J. L. Collier, K. Coyne, S. K. Davy, P. Deschamps, S. T. Dyhrman, B. Edvardsen, R. D. Gates, C. J. Gobler, S. J. Greenwood, S. M. Guida, J. L. Jacobi, K. S. Jakobsen, E. R. James, B. Jenkins, U. John, M. D. Johnson, A. R. Juhl, A. Kamp, L. A. Katz, R. Kiene, A. Kudryavtsev, B. S. Leander, S. Lin, C. Lovejoy, D. Lynn, A. Marchetti, G. McManus, A. M. Nedelcu, S. Menden-Deuer, C. Miceli, T. Mock, M. Montresor, M. A. Moran, S. Murray, G. Nadathur, S. Nagai, P. B. Ngam, B. Palenik, J. Pawlowski, G. Petroni, G. Piganeau, M. C. Posewitz, K. Rengefors, G. Romano, M. E. Rumpho, T. Rynearson, K. B. Schilling, D. C. Schroeder, A. G. B. Simpson, C. H. Slamovits, D. R. Smith, G. J. Smith, S. R. Smith, H. M. Sosik, P. Stief, E. Theriot, S. Twary, P. E. Umale, D. Vaulot, B. Wawrik, G. L. Wheeler, W. H. Wilson, Y. Xu, A. Zingone, and A. Z. Worden. 2014. The Marine Microbial Eukaryote Transcriptome Sequencing Project (MMETSP): Illuminating the functional diversity of eukaryotic life in the oceans through transcriptome sequencing. PLoS. Biol. 12:e1001889.

Ki, J.-S. 2010. Nuclear 28S rDNA phylogeny supports the basal placement of *Noctiluca scintillans* (Dinophyceae; Noctilucales) in dinoflagellates. European Journal of Protistology 46:111–120.

Ki, J.-S., and M.-S. Han. 2005. Efficient 5'ETS walking from conserved 18S rDNA sequences of the dinoflagellates *Alexandrium* and *Akashiwo sanguinea* (Dinophyceae). Journal of Applied Phycology 17:475–481.

Ki, J.-S., and M.-S. Han. 2007a. Cryptic long internal repeat sequences in the ribosomal DNA ITS1 gene of the dinoflagellate *Cochlodinium polykrikoides* (Dinophyceae): A 101 nucleotide six-repeat track with a palindrome-like structure. Genes & Genetic Systems 82:161–166.

Ki, J.-S., and M.-S. Han. 2007b. Informative characteristics of 12 divergent domains in complete large subunit rDNA sequences from the harmful dinoflagellate genus, *Alexandrium* (Dinophyceae). Journal of Eukaryotic Microbiology 54:210–219.

Ki, J.-S., and M.-S. Han. 2007c. Rapid molecular identification of the harmful freshwater dinoflagellate *Peridinium* in various life stages using genus-specific single-cell PCR. Journal of Applied Phycology 19:467–470.

Ki, J.-S., M.-H. Park, and M.-S. Han. 2011. Discriminative power of nuclear rDNA sequences for the DNA taxonomy of the dinoflagellate genus *Peridinium* (Dinophyceae). Journal of Phycology 47:426–435.

Kremp, A., M. Elbrächter, M. Schweikert, J. L. Wolny, and M. Gottschling. 2005. *Woloszynskia halophila* (Biecheler) comb. nov.: A bloom-forming cold-water dinoflagellate co-occurring with *Scrippsiella hangoei* (Dinophyceae) in the Baltic Sea. Journal of Phycology 41:629–642.

Kretschmann, J., N. H. Filipowicz, P. M. Owsianny, C. Zinßmeister, and M. Gottschling. 2015. Taxonomic clarification of the unusual dinophyte *Gymnodinium limneticum* Wołosz. (Gymnodiniaceae) from the Tatra Mountains. Protist 166:621–637.

Kretschmann, J., P. M. Owsianny, A. Žerdoner Čalasan, and M. Gottschling. 2018a. The hot spot in a cold environment: Puzzling *Parvodinium* (Peridiniopsidaceae, Peridiniales) from the Polish Tatra Mountains. Protist 169:206–230.

Kretschmann, J., A. Žerdoner Čalasan, and M. Gottschling. 2018b. Molecular phylogenetics of dinophytes harbouring diatoms as endosymbionts (Kryptoperidiniaceae, Peridiniales), with evolutionary interpretations and a focus on the identity of *Durinskia oculata* from Prague. Molecular Phylogenetics and Evolution 118:392–402.

Kretschmann, J., A. Žerdoner Čalasan, W.-H. Kusber, and M. Gottschling. 2018c. Still curling after all these years: *Glenodinium apiculatum* Ehrenb. (Peridiniales, Dinophyceae) repeatedly found at its type locality in Berlin (Germany). Systematics and Biodiversity 16:200–209.

Leblond, J. D., A. D. Lasiter, C. Li, R. Logares, K. Rengefors, and T. J. Evens. 2010. A data mining approach to dinoflagellate clustering according to sterol composition: Correlations with evolutionary history. International Journal of Data Mining and Bioinformatics 4:431–451.

Lin, S., H. Zhang, Y. Hou, L. Miranda, and D. Bhattacharya. 2006a. Development of a dinoflagellate-oriented PCR primer set leads to detection of picoplanktonic dinoflagellates from Long Island Sound. Applied and Environmental Microbiology 72:5626–5630.

Lin, S., H. Zhang, and N. Z. Jiao. 2006b. Potential utility of mitochondrial cytochrome b and ITS mRNA editing in resolving closely related dinoflagellates: A case study of *Prorocentrum* (Dinophyceae). Journal of Phycology 42:646–654.

Lindberg, K., Ø. Moestrup, and N. Daugbjerg. 2005. Studies on woloszynskioid dinoflagellates - I: *Woloszynskia coronata* re-examined using light and electron microscopy and partial LSU rDNA sequences, with description of *Tovellia* gen. nov. and *Jadwigia* gen. nov. (Tovelliaceae fam. nov.). Phycologia 44:416–440.

Logares, R., A. Boltovskoy, S. Bensch, J. Laybourn-Parry, and K. Rengefors. 2009. Genetic diversity patterns in five protist species occurring in lakes. Protist 160:301–317.

Logares, R., N. Daugbjerg, A. Boltovskoy, A. Kremp, J. Laybourn-Parry, and K. Rengefors. 2008. Recent evolutionary diversification of a protist lineage. Environmental Microbiology 10:1231–1243.

Logares, R., K. Rengefors, A. Kremp, K. Shalchian-Tabrizi, A. Boltovskoy, T. Tengs, A. Shurtleff, and D. Klaveness. 2007a. Phenotypically different microalgal morphospecies with identical ribosomal DNA: A case of rapid adaptive evolution? Microbial Ecology 53:549–561.

Logares, R., K. Shalchian-Tabrizi, A. Boltovskoy, and K. Rengefors. 2007b. Extensive dinoflagellate phylogenies indicate infrequent marine-freshwater transitions. Molecular Phylogenetics and Evolution 45:887–903.

Luo, Z., B. Krock, K. N. Mertens, E. Nézan, N. Chomérat, G. Billen, U. Tillmann, and H. Gu. 2017. Adding new pieces to the *Azadinium* (Dinophyceae) diversity and biogeography puzzle: Non-toxigenic *Azadinium zhuanum* sp. nov. from China, toxigenic *A. poporum* from the Mediterranean, and a non-toxigenic *A. dalianense* from the French Atlantic. Harmful Algae 66:65–78.

Luo, Z., Z. F. Lim, K. N. Mertens, P. Gurdebeke, K. Bogus, M. C. Carbonell-Moore, H. Vrielinck, C. P. Leaw, P. T. Lim, N. Chomérat, X. Li, and H. Gu. 2018. Morpho-molecular diversity and phylogeny of *Bysmatrum* (Dinophyceae) from the South China Sea and France. European Journal of Phycology 53:318–335.

Luo, Z., Mertens, K.N., Nézan, E., Gu, L., Pospelova, V., Thoha, H., Gu, H., 2019. Morphology, ultrastructure and molecular phylogeny of cyst-producing *Caladoa arcachonensis* gen. et sp. nov. (Peridiniales, Dinophyceae) from France and Indonesia. European Journal of Phycology 54, 235–248.

Luo, Z., X. You, K. N. Mertens, and H. Gu. 2016. Morphological and molecular characterization of *Tovellia* cf. *aveirensis* (Dinophyceae) from Jiulong River, China. Nova Hedwigia 103:79–94.

Massana, R., B. Karniol, T. Pommier, I. Bodaker, and O. Beja. 2008. Metagenomic retrieval of a ribosomal DNA repeat array from an uncultured marine alveolate. Environmental Microbiology 10:1335–1343.

Matsuoka, K., and H. Kawami. 2013. Phylogenetic subdivision of the genus *Protoperidinium* (Peridiniales, dinophyceae) with emphasis on the *Monovela* group. Pp. 275-284 *in* J. Lewis, F. Marret, and L. Bradley, eds. Biological and geological perspectives of dinoflagellates. Micropalaeontological Society by the Geological Society, London.

McCauley, L. A. R., D. L. Erdner, S. Nagai, M. L. Richlen, and D. M. Anderson. 2009. Biogeographic analysis of the globally distributed algal bloom species *Alexandrium minutum* (Dinophyceae) based on rRNA gene sequences and microsatellite markers. Journal of Phycology 45:454–463.

Mertens, K. N., M. Bringue, N. Van Nieuwenhove, Y. Takano, V. Pospelova, A. Rochon, A. De Vernal, T. Radi, B. Dale, R. T. Patterson, K. Weckstrom, E. Andren, S. Louwye, and K. Matsuoka. 2012. Process length variation of the cyst of the dinoflagellate *Protoceratium reticulatum* in the North Pacific and Baltic-Skagerrak region: Calibration as an annual density proxy and first evidence of pseudo-cryptic speciation. Journal of Quaternary Science 27:734–744.

Mertens, K. N., Y. Takano, H. Gu, S. Bagheri, V. Pospelova, A. J. Pienkowski, S. A. G. Leroy, and K. Matsuoka. 2017. Cyst-theca relationship and phylogenetic position of *Impagidinium caspienense* incubated from Caspian Sea surface sediments: Relation to *Gonyaulax baltica* and evidence for heterospory within gonyaulacoid dinoflagellates. Journal of Eukaryotic Microbiology 64:829–842.

Moestrup, Ø., and N. Daugbjerg. 2007. On dinoflagellate phylogeny and classification. Pp. 215–230 *in* J. Brodie, and J. Lewis, eds. Unravelling the algae, the past, present, and future of algal systematics. CRC Press, Boca Raton.

Moestrup, Ø., K. H. Nicholls, and N. Daugbjerg. 2018. Studies on woloszynskioid dinoflagellates IX: Ultrastructure, cyst formation and phylogeny of the 'red-snow' alga *Borghiella pascheri*

(Suchlandt) Moestrup (= *Glenodinium pascheri*, *Woloszynskia pascheri*, *Gyrodinium nivalis*). European Journal of Phycology 53:393–409.

Montresor, M., S. Sgrosso, G. Procaccini, and W. H. C. F. Kooistra. 2003. Intraspecific diversity in *Scrippsiella trochoidea* (Dinophyceae): Evidence for cryptic species. Phycologia 42:56–70.

Murray, S. A., C. L. C. Ip, R. Moore, Y. Nagahama, and Y. Fukuyo. 2009. Are prorocentroid dinoflagellates monophyletic? A study of 25 species based on nuclear and mitochondrial genes. Protist 160:245–264.

Murray, S. A., M. F. Jørgensen, N. Daugbjerg, and L. Rhodes. 2004. *Amphidinium* revisited. II. Resolving species boundaries in the *Amphidinium operculatum* species complex (Dinophyceae), including the descriptions of *Amphidinium trulla* sp. nov. and *Amphidinium gibbosum* comb. nov. Journal of Phycology 40:366–382.

Nagahama, Y., S. A. Murray, A. Tomaru, and Y. Fukuyo. 2011. Species boundaries in the toxic dinoflagellate *Prorocentrum lima* (Dinophyceae, Prorocentrales), based on morphological and phylogenetic characters. Journal of Phycology 47:178–189.

Nézan, E., R. Siano, S. Boulben, C. Six, G. Bilien, K. Cheze, A. Duval, S. Le Panse, J. Quéré, and N. Chomérat. 2014. Genetic diversity of the harmful family Kareniaceae (Gymnodiniales, Dinophyceae) in France, with the description of *Karlodinium gentienii* sp. nov.: A new potentially toxic dinoflagellate. Harmful Algae 40:75–91.

Nézan, E., U. Tillmann, G. Bilien, S. Boulben, K. Chèze, F. Zentz, R. Salas, and N. Chomérat. 2012. Taxonomic revision of the dinoflagellate *Amphidoma caudata*: Transfer to the genus *Azadinium* (Dinophyceae) and proposal of two varieties, based on morphological and molecular phylogenetic analyses. Journal of Phycology 48:925–939.

Orr, R. J. S., S. A. Murray, A. Stüken, L. Rhodes, and K. S. Jakobsen. 2012. When naked became armored: An eight-gene phylogeny reveals monophyletic origin of theca in dinoflagellates. PLoS One 7:e50004.

Pecher, W. T., J. A. F. Robledo, and G. R. Vasta. 2004. Identification of a second rRNA gene unit in the *Perkinsus andrewsi* genome. Journal of Eukaryotic Microbiology 51:234–245.

Pochon, X., J. Pawlowski, L. Zaninetti, and R. Rowan. 2001. High genetic diversity and relative specificity among *Symbiodinium*-like endosymbiotic dinoflagellates in soritid foraminiferans. Marine Biology 139:1069–1078.

Pochon, X., H. M. Putnam, F. Burki, and R. D. Gates. 2012. Identifying and characterizing alternative molecular markers for the symbiotic and free-living dinoflagellate genus *Symbiodinium*. PLoS One 7:e29816.

Potvin, É., S.-Y. Kim, E. J. Yang, M. J. Head, H.-c. Kim, S.-I. Nam, J. H. Yim, and S.-H. Kang. 2018. *Islandinium minutum* subsp. *barbatum* subsp. nov. (Dinoflagellata), a new organic-walled dinoflagellate cyst from the Western Arctic: Morphology, phylogenetic position based on SSU rDNA and LSU rDNA, and distribution. Journal of Eukaryotic Microbiology 65:750–772.

Prabowo, D. A., M. M. R. Shah, T. Horiguchi, and S. Suda. 2016. Genetic diversity of *Moestrupia oblonga* (Dinophyceae) from coastal areas of Okinawa Island, Japan. Marine Biodiversity 46:197–209.

Robledo, J. A. F., P. A. Nunes, M. L. Cancela, and G. R. Vasta. 2002. Development of an in vitro clonal culture and characterization of the rRNA gene cluster of *Perkinsus atlanticus*, a protistan parasite of the clam *Tapes decussatus*. Journal of Eukaryotic Microbiology 49:414–422.

Rogers, J. E., J. D. Leblond, and C. A. Moncreiff. 2006. Phylogenetic relationship of *Alexandrium monilatum* (Dinophyceae) to other *Alexandrium* species based on 18S ribosomal RNA gene sequences. Harmful Algae 5:275–280.

Romeikat, C., J. Knechtel, and M. Gottschling. 2020. Clarifying the taxonomy of *Gymnodinium fuscum* var. *rubrum* from Bavaria (Germany) and placing it in a molecular phylogeny of the Gymnodiniaceae (Dinophyceae). Systematics and Biodiversity 18:102–115.

Saito, K., T. Drgon, J. A. F. Robledo, D. N. Krupatkina, and G. R. Vasta. 2002. Characterization of the rRNA locus of *Pfiesteria piscicida* and development of standard and quantitative PCR-based detection assays targeted to the nontranscribed spacer. Applied and Environmental Microbiology 68:5394–5407.

Saldarriaga Echavarría, J. F., M. L. McEwan, N. M. Fast, F. J. R. Taylor, and P. J. Keeling. 2003. Multiple protein phylogenies show that *Oxyrrhis marina* and *Perkinsus marinus* are early branches of the dinoflagellate lineage. International Journal of Systematic and Evolutionary Microbiology 53:355–365.

Saldarriaga Echavarría, J. F., F. J. R. Taylor, T. Cavalier-Smith, S. Menden-Deuerd, and P. J. Keeling. 2004. Molecular data and the evolutionary history of dinoflagellates. European Journal of Protistology 40:85–111.

Saldarriaga Echavarría, J. F., F. J. R. Taylor, P. J. Keeling, and T. Cavalier-Smith. 2001. Dinoflagellate nuclear SSU rRNA phylogeny suggests multiple plastid losses and replacements. Journal of Molecular Evolution 53:204–213.

Saunders, G. W., D. R. A. Hill, J. P. Sexton, and R. A. Andersen. 1997. Small-subunit ribosomal RNA sequences from selected dinoflagellates: Testing classic evolutionary hypotheses with molecular systematic methods. Plant Systematics and Evolution (Supplement) 11:237–259.

Scorzetti, G., L. E. Brand, G. L. Hitchcock, K. S. Rein, C. D. Sinigalliano, and J. W. Fell. 2009. Multiple simultaneous detection of Harmful Algal Blooms (HABs) through a high throughput bead array technology, with potential use in phytoplankton community analysis. Harmful Algae 8:196–211.

Selina, M. S., K. V. Efimova, and M. Hoppenrath. 2019. Redefinition of the genus *Thecadinium* (Dinophyceae) using morphological and molecular data, and description of *Thecadinium pseudokofoidii* sp. nov. Phycologia 58:36–50.

Shoguchi, E., C. Shinzato, T. Kawashima, F. Gyoja, S. Mungpakdee, R. Koyanagi, T. Takeuchi, K. Hisata, M. Tanaka, M. Fujiwara, M. Hamada, A. Seidi, M. Fujie, T. Usami, H. Goto, S. Yamasaki, N. Arakaki, Y. Suzuki, S. Sugano, A. Toyoda, Y. Kuroki, A. Fujiyama, M. Medina, M. A. Coffroth, D. Bhattacharya, and N. Satoh. 2013. Draft assembly of the *Symbiodinium minutum* nuclear genome reveals dinoflagellate gene structure. Current Biology 23:1399–1408.

Siano, R., W. H. C. F. Kooistra, M. Montresor, and A. Zingone. 2009. Unarmoured and thin-walled dinoflagellates from the Gulf of Naples, with the description of *Woloszynskia cincta* sp. nov. (Dinophyceae, Suessiales). Phycologia 48:44–65.

Sparmann, S. F., B. S. Leander, and M. Hoppenrath. 2008. Comparative morphology and molecular phylogeny of *Apicoporus* n. gen.: A new genus of marine benthic dinoflagellates formerly classified within *Amphidinium*. Protist 159:383–399.

Stern, R. F., R. A. Andersen, I. Jameson, F. C. Küpper, M.-A. Coffroth, D. Vaulot, F. Le Gall, B. Véron, J. J. Brand, H. Skelton, F. Kasai, E. L. Lilly, and P. J. Keeling. 2012. Evaluating the ribosomal Internal Transcribed Spacer (ITS) as a candidate dinoflagellate barcode marker. PLoS One 7:e42780.

Stern, R. F., A. Horak, R. L. Andrew, M.-A. Coffroth, R. A. Andersen, F. C. Küpper, I. Jameson, M. Hoppenrath, B. Véron, F. Kasai, J. Brand, E. R. James, and P. J. Keeling. 2010. Environmental barcoding reveals massive dinoflagellate diversity in marine environments. PLoS One 5:e13991.

Takahashi, K., Ø. Moestrup, R. W. Jordan, and M. Iwataki. 2015. Two new freshwater woloszynskioids *Asulcocephalium miricentonis* gen. et sp. nov. and *Leiocephalium*

pseudosanguineum gen. et sp. nov. (Suessiaceae, Dinophyceae) lacking an apical furrow apparatus. Protist 166:638–658.

Takahashi, K., Ø. Moestrup, M. Wada, A. Ishimatsu, N. Van Nguyen, Y. Fukuyo, and M. Iwataki. 2017. *Dactylodinium pterobelotum* gen. et sp. nov., a new marine woloszynskioid dinoflagellate positioned between the two families Borghiellaceae and Suessiaceae. Journal of Phycology 53:1223–1240.

Takahashi, K., Benico, G., Lum, W.M., Iwataki, M., in press. *Gertia stigmatica* gen. et sp. nov. (Kareniaceae, Dinophyceae), a new marine unarmored dinoflagellate possessing the peridinin-type chloroplast with an eyespot. Protist 170.

Takano, Y., and T. Horiguchi. 2006. Acquiring scanning electron microscopical, light microscopical and multiple gene sequence data from a single dinoflagellate cell. Journal of Phycology 42:251–256.

Takano, Y., H. Yamaguchi, I. Inouye, Ø. Moestrup, and T. Horiguchi. 2014. Phylogeny of five species of *Nusuttodinium* gen. nov. (Dinophyceae), a genus of unarmoured kleptoplastidic dinoflagellates. Protist 165:759–778.

Tang, X., R. Yu, M. Zhou, and Z. Yu. 2012. Application of rRNA probes and fluorescence in situ hybridization for rapid detection of the toxic dinoflagellate *Alexandrium minutum*. Journal of Oceanology and Limnology 30:256–263.

Thomson, P. G., S. W. Wright, C. J. S. Bolch, P. D. Nichols, J. H. Skerratt, and A. McMinn. 2004. Antarctic distribution, pigment and lipid composition, and molecular identification of the brine dinoflagellate *Polarella glacialis* (Dinophyceae). Journal of Phycology 40:867–873.

Tillmann, U., M. Gottschling, E. Nézan, and B. Krock. 2015. First records of *Amphidoma languida* and *Azadinium dexteroporum* (Amphidomataceae, Dinophyceae) from the Irminger Sea off Iceland. Marine Biodiversity Records 8:e142.

Tillmann, U., M. Gottschling, E. Nézan, B. Krock, and G. Bilien. 2014. Morphological and molecular characterization of three new *Azadinium* species (Amphidomataceae, Dinophyceae) from the Irminger Sea. Protist 165:417–444.

Tillmann, U., M. Hoppenrath, M. Gottschling, W.-H. Kusber, and M. Elbrächter. 2017. Plate pattern clarification of the marine dinophyte *Heterocapsa triquetra sensu* Stein (Dinophyceae) collected at the Kiel Fjord (Germany). Journal of Phycology 53:1305–1324.

Tillmann, U., S. Söhner, E. Nézan, and B. Krock. 2012. First record of the genus *Azadinium* (Dinophyceae) from the Shetland Islands, including the description of *Azadinium polongum* sp. nov. Harmful Algae 20:142–155.

Wang, N., Z. Luo, K. N. Mertens, F. M. G. McCarthy, L. Gu, and H. Gu. 2017. Cyst-motile stage relationship and molecular phylogeny of a new freshwater dinoflagellate *Gymnodinium plasticum* from Plastic Lake, Canada. Phycological Research 65:312–321.

Watanabe, K., Y. Miyoshi, F. Kubo, R. Onuma, S. A. Murray, and T. Horiguchi. 2014. *Ankistrodinium armigerum* sp. nov. (Dinophyceae), a new species of heterotrophic marine sand-dwelling dinoflagellate from Japan and Australia. Phycological Research 62:125–135.

Yamada, N., R. Terada, A. Tanaka, and T. Horiguchi. 2013. *Bispinodinium angelaceum* gen. et sp. nov. (Dinophyceae), a new sand-dwelling dinoflagellate from the seafloor off Mageshima Island, Japan. Journal of Phycology 49:555–569.

Yamaguchi, A., M. Hoppenrath, V. Pospelova, T. Horiguchi, and B. S. Leander. 2011. Molecular phylogeny of the marine sand-dwelling dinoflagellate *Herdmania litoralis* and an emended description of the closely related planktonic genus *Archaeperidinium* Jörgensen. European Journal of Phycology 46:98–112.

Yamaguchi, A., and T. Horiguchi. 2005. Molecular phylogenetic study of the heterotrophic dinoflagellate genus *Protoperidinium* (Dinophyceae) inferred from small subunit rRNA gene sequences. Phycological Research 53:30–42.

Yamaguchi, A., H. Kawamura, and T. Horiguchi. 2006. A further phylogenetic study of the heterotrophic dinoflagellate genus, *Protoperidinium* (Dinophyceae) based on small and large subunit ribosomal RNA gene sequences. Phycological Research 54:317–329.

Yamaguchi, A., H. Kawamura, and T. Horiguchi. 2007. The phylogenetic position of an unusual *Protoperidinium* species, *P. bipes* (Peridiniales, Dinophyceae), based on small and large subunit ribosomal RNA gene sequences. Phycologia 46:270–276.

Yamaguchi, A., S. Yoshimatsu, M. Hoppenrath, K. C. Wakeman, and H. Kawai. 2016. Molecular phylogeny of the benthic dinoflagellate genus *Amphidiniopsis* and its relationships with the family Protoperidiniaceae. Protist 167:568–583.

Yoon, E. Y., N. S. Kang, and H. J. Jeong. 2012. *Gyrodinium moestrupii* n. sp., a new planktonic heterotrophic dinoflagellate from the coastal waters of Western Korea: Morphology and ribosomal DNA gene sequence. Journal of Eukaryotic Microbiology 59:571–586.

Yoshida, T., R. Nakai, H. Seto, M. K. Wang, M. Iwataki, and S. Hiroishi. 2003. Sequence analysis of 5.8S rDNA and the Internal Transcribed Spacer region in dinoflagellate *Heterocapsa* species (Dinophyceae) and development of selective PCR primers for the bivalve killer *Heterocapsa circularisquama*. Microbes and Environments 18:216–222.

Žerdoner Čalasan, A., J. Kretschmann, and M. Gottschling. 2018. Absence of co-phylogeny indicates repeated diatom capture in dinophytes hosting a tertiary endosymbiont. Organisms Diversity & Evolution 18:29–38.

Žerdoner Čalasan, A., J. Kretschmann, and M. Gottschling. 2019. They are young, and they are many: Dating freshwater lineages in unicellular dinophytes. Environmental Microbiology 21:4125–4135.

Zhang, B.-Y., G.-C. Wang, Y. Zhang, X.-T. Han, S.-H. Lo, Y.-Z. Qi, J.-Z. Zou, and C.-K. Zeng. 2004. 东海原甲藻 (*Prorocentrum donghaiense*) 和海洋原甲藻 APBM (*P.micans* APBM) 的 5.8S rDNA 及其转录间隔区 (ITS) 的克隆和序列分析. Oceanologia et limnologia sinica 35:265–272.

Zhang, H., D. Bhattacharya, L. Maranda, and S. Lin. 2008. Mitochondrial *cob* and *cox1* genes and editing of the corresponding mRNAs in *Dinophysis acuminata* from Narragansett Bay, with special reference to the phylogenetic position of the genus *Dinophysis*. Applied and Environmental Microbiology 74:1546–1554.

Zhang, Q., G.-X. Liu, and Z.-Y. Hu. 2011. Morphological differences and molecular phylogeny of freshwater blooming species, *Peridiniopsis* spp. (Dinophyceae) from China. European Journal of Protistology 47:149–160.

Zhang, W., Z. Li, K. N. Mertens, A. Derrien, V. Pospelova, M. C. Carbonell-Moore, S. Bagheri, K. Matsuoka, H. H. Shin, and H. Gu. 2020. Reclassification of *Gonyaulax verior* (Gonyaulacales, Dinophyceae) as *Sourniaea diacantha gen. et comb. nov.* Phycologia 59:246–260.

Zhuang, L., Y.-Q. Chen, Q.-L. Li, and L.-H. Qu. 2001. **赤潮叉角藻18SrDNA和ITS区序列**测定与分析. Oceanologia et limnologia sinica 32:148–154.

Zinßmeister, C., S. Söhner, M. Kirsch, E. Facher, K. J. S. Meier, H. Keupp, and M. Gottschling. 2012. Same but different: Two novel bicarinate species of extant calcareous dinophytes (Thoracosphaeraceae, Peridiniales) from the Mediterranean Sea. Journal of Phycology 48:1107–1118.
